# Mitochondrial and Proteasome Dysfunction Occurs in the Hearts of Mice Treated with Triazine Herbicide Prometryn

**DOI:** 10.3390/ijms242015266

**Published:** 2023-10-17

**Authors:** Rasheed O. Sule, Brett S. Phinney, Michelle R. Salemi, Aldrin V. Gomes

**Affiliations:** 1 Department of Neurobiology, Physiology, and Behavior, University of California, Davis, One Shields Ave., Davis, CA 95616, USA; 2 Center for Mitochondrial and Epigenomic Medicine, Children’s Hospital of Philadelphia, Philadelphia, PA 19104, USA; 3 Proteomics Core Facility, University of California, Davis, Davis, CA 95616, USA; bsphinney@ucdavis.edu (B.S.P.); msalemi@ucdavis.edu (M.R.S.); 4 Department of Physiology and Membrane Biology, University of California, Davis, Davis, CA 95616, USA

**Keywords:** prometryn, proteomics, mitochondria, oxidative stress, heart, cardiovascular diseases, metabolism, pesticides, toxicity, antioxidant

## Abstract

Prometryn is a methylthio-s-triazine herbicide used to control the growth of annual broadleaf and grass weeds in many cultivated plants. Significant traces of prometryn are documented in the environment, mainly in waters, soil, and plants used for human and domestic consumption. Previous studies have shown that triazine herbicides have carcinogenic potential in humans. However, there is limited information about the effects of prometryn on the cardiac system in the literature, or the mechanisms and signaling pathways underlying any potential cytotoxic effects are not known. It is important to understand the possible effects of exogenous compounds such as prometryn on the heart. To determine the mechanisms and signaling pathways affected by prometryn (185 mg/kg every 48 h for seven days), we performed proteomic profiling of male mice heart with quantitative liquid chromatography-tandem mass spectrometry (LC-MS/MS) using ten-plex tandem mass tag (TMT) labeling. The data suggest that several major pathways, including energy metabolism, protein degradation, fatty acid metabolism, calcium signaling, and antioxidant defense system were altered in the hearts of prometryn-treated mice. Proteasome and immunoproteasome activity assays and expression levels showed proteasome dysfunction in the hearts of prometryn-treated mice. The results suggest that prometryn induced changes in mitochondrial function and various signaling pathways within the heart, particularly affecting stress-related responses.

## 1. Introduction

Pesticides are a group of chemicals, and sometimes microorganisms (e.g., viruses), that are commonly used for the eradication of insects, weeds, fungi, and bacteria [1]. The broad use of pesticides for industrial, commercial, and individual households indicates the importance of these compounds to humans, plants, and even animals [1]. Many of the applied herbicides are bioactive compounds that are relatively water soluble and thus can be transported to the bodies of surface water or groundwater resources [2]. Pesticides primarily enter the surface and groundwater as run-off from agricultural field and industrial wastewater [2]. The direct and indirect contamination of surface waters by pesticides is known to occur via aerial drift, watershed run-off, and accidental spillage during the widespread use of these chemicals in agriculture and forestry [3].

Triazine herbicides belong to a category classified as persistent organic compounds since they resist biological and chemical degradation [4]. 2,4-Bis (isopropylamino)-6-methylthio-1,3,5-triazine (prometryn) is a widely used selective triazine herbicide, which is currently used as an alternative to atrazine-based products. This bioactive compound is widely applied in agriculture due to its lower acute toxicity when compared to other atrazine herbicides. Another advantage of using prometryn is its stable chemical properties and a prolonged period of effectiveness [5]. However, prometryn is relatively persistent in waters, soil, and even in the air near its production or application sites, and it has been detected at a concentration of 3–6.1 μg/L in different rivers and lakes in Europe [1]. We and other researchers have previously published studies that highlight the high amount of triazine herbicides found in groundwater, surface waters, river basins in agricultural areas in Europe and North America [1,6,7]. Navarro et al. showed that four s-triazine herbicides, including prometryn, were persistent in river, sea, and groundwater samples exposed to sunlight and darkness under laboratory conditions and required about 100–336 days to degrade, which highlighted their long half-lives and the compound’s persistence in different environments [8].

A common feature of pesticides that was observed in different cells and animal tissues was the induction of oxidative stress which mostly resulted in lipid oxidation, DNA oxidation, and protein oxidation [1]. Exposure to different classes of pesticides could lead to net production of reactive oxygen species (ROS), thereby inducing oxidative stress beyond what the intrinsic cellular antioxidant system can mitigate to normal physiological levels due to being overwhelmed [9]. Mitochondria are the primary site for ROS production as a by-product of their role in energy generation. It was discovered that mitochondrial ROS (mtROS) directly stimulate the production of proinflammatory cytokines. Pathological conditions such as autoimmune diseases and cardiovascular diseases all share a common phenotype of increased mtROS production above the basal level [10]. Indeed, it is estimated that about 30–40% of mitochondria occupy the cardiomyocytes’ volume [11]. With this substantial amount of mitochondria, the heart is particularly more susceptible to damage through oxidative stress, which can result in a plethora of cardiovascular complications [12]. In a recent investigation involving marine medaka (*Oryzias melastigma*) embryos, exposure to varying concentrations of prometryn was found to have notable effects [13]. The study revealed that even at very low concentrations of prometryn, as little as 1 µg/L, there were significant delays in hatching time, increased heart rates, and a higher incidence of hatching failure among the embryos. Consequently, the study’s findings suggest that environmentally relevant levels of prometryn can induce substantial toxicity during the early developmental stages of this species of fish. Notably, the cytotoxic effects of prometryn on the heart and the mechanisms underlying these effects, to our knowledge, has not been previously researched. Therefore, it is particularly important to investigate the mechanisms and signaling pathways associated with hearts from prometryn-treated mice.

To investigate the mechanisms by which prometryn affects mouse heart, mice were treated with prometryn and their hearts were compared to vehicle-treated mice via a quantitative LC-MS/MS technique using tandem mass tag (TMT) labeling. In vitro studies were also carried out to determine the effect of prometryn on ROS formation and mitochondrial function. We tested the hypothesis that treatment with prometryn would increase oxidative stress and decrease proteasome and mitochondria function. Prometryn was found to alter proteins involved in pathways such as glycolysis, protein degradation, fatty acid metabolism, and the antioxidant system. Biological assays and immunoblotting demonstrated significant differences in the heart from prometryn-treated groups relative to their respective controls.

## 2. Results

### 2.1. Effects of Prometryn on H9c2 Cardiac Cells

#### 2.1.1. Prometryn Treatment Reduces Cell Viability in H9c2 Cells

**Figure 1:**
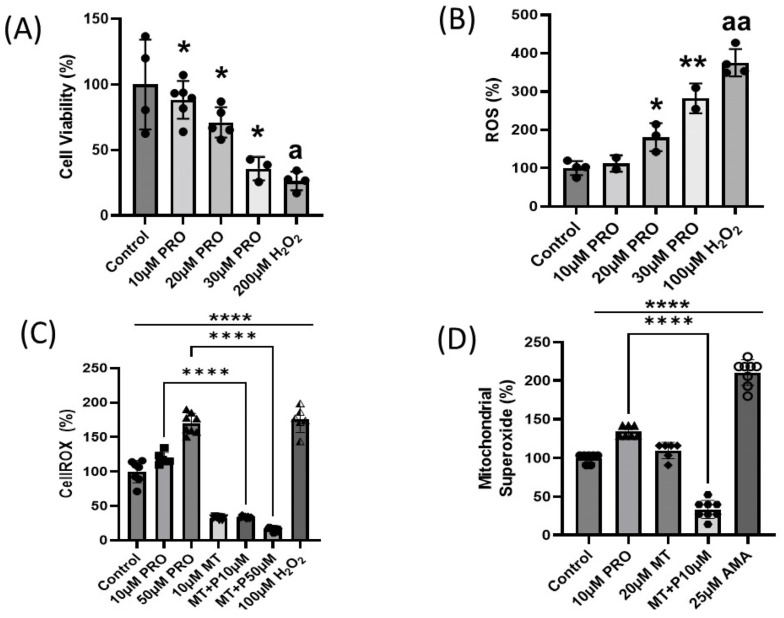
Concentration-dependent cytotoxicity of prometryn in H9c2 cardiac cells. (**A**) H9c2 cells were treated with prometryn (0–30 μM) for 24 h, and cell viability was measured using AlamarBlue. (**B**) ROS formation was detected in H9c2 cells stained with a cell permeable fluorescent probe H_2_DCFDA and treated with 10–30 μM prometryn. H_2_O_2_ (200 μM) was used as a positive oxidative stress control. ROS levels were detected via fluorescence spectroscopy at Ex-502 nm and Em-523 nm. (**C**) Increased oxidative stress in H9c2 cardiac cells treated with prometryn was confirmed via staining with CellROX Green. Meanwhile, 1 h pretreatment with MitoTEMPO significantly decreased the oxidative stress. (**D**) Superoxide anion levels increased after treatment with prometryn. However, mitochondrial-targeted antioxidant MitoTEMPO significantly decreased the superoxide level in the presence of prometryn in H9c2 cardiac cells. Spectrofluorometric data expressing the fluorescence intensity of MitoSOX after treatment with prometryn and/or MitoTEMPO are shown. PRO = Prometryn, MT = MitoTEMPO, MT + P = MitoTEMPO + Prometryn, AMA = Antimycin A. Values are expressed as mean ± SD (*n* ≥ 3). * *p* < 0.05, ** *p* < 0.001, **** *p* < 0.0001. (**A**) ^a^ *p* < 0.05 Control vs. 200 μM H_2_O_2_, (**B**) ^aa^ *p* < 0.001 Control vs. 100 μM H_2_O_2_.

A previous study found that triazine pesticides such as atrazine, the same class of pesticides that prometryn belongs to, induces cell death via apoptosis in different cell types and tissues [14]. To determine if prometryn has cytotoxic effects on cardiac cells in vitro, cell viability was determined with the alamarBlue assay. After 24 h exposure to different concentrations of prometryn (10–30 μM), we observed a significant concentration-dependent decrease in the number of viable cells (Figure 1A). Data are expressed as relative to the controls (0.1% DMSO).

#### 2.1.2. Prometryn Treatment Elevated Oxidative Stress and Mitochondrial Superoxide

Since decreased cell viability was observed after prometryn treatment, it was important to establish the factors contributing to cell death. Therefore, we investigated whether prometryn affects ROS levels in H9c2 cells. The cells were stained with the fluorescent probe DCFH-DA and CellROX, and the changes in intracellular ROS levels were quantitatively detected via fluorescence intensity. We found that prometryn treatment resulted in an elevated ROS generation in a concentration-dependent manner. Note that 100 μM H_2_O_2_ was used as a positive control for ROS formation (Figure 1B,C). Our data suggest that prometryn induced ROS generation in H9c2 cells even at low concentrations such as 20 and 30 μM. Increased mitochondrial superoxide anion could be one of the ways ROS is produced after prometryn treatment. The mitochondrial superoxide level was determined after treating H9c2 cells with different concentrations of prometryn. As an added control, parallel experiments were conducted where H9c2 cells were also treated with a mitochondrial superoxide scavenger MitoTEMPO. Using MitoSOX Red mitochondrial superoxide indicator as a probe, we observed a significant elevation in the mitochondrial superoxide anion in prometryn-treated H9c2 cardiac cells (Figure 1D). When MitoTEMPO (10 μM) was added with prometryn, it prevented the increased levels of mitochondrial superoxide caused by prometryn alone. Antimycin A was used as positive control because of the ability to induce superoxide ions in mitochondria [15]. This data showed that prometryn induces intracellular ROS and mitochondrial superoxide, suggesting that oxidative stress is increased in prometryn treated cardiac cells.

### 2.2. Identification of the Differentially Expressed Proteins in Prometryn Treated Mice

Since prometryn induced significant ROS and decreased cell viability at the in vitro level, we sought to understand the effects of prometryn treatment in vivo using 10-week-old male mice. We employed proteomics based on mass spectrometry (MS) which allows us to identify, detect, and characterize protein expression and function on a wide scale with exquisite precision and sensitivity [16]. Proteomic profiling with quantitative 10-plex TMT LC-MS/MS showed significant differences between hearts of vehicle-treated and prometryn-treated mice. This is the first report on proteomic profiling of hearts from mice treated with prometryn. A total of 264 differentially expressed proteins were identified and filtered based on species “*Mus musculus*”. The upregulated and downregulated proteins were grouped by biological process using PANTHER Go-Slim program option with a *p*-value of <0.005 and an FDR of <0.5%, as shown in Table 1.

### 2.3. Gene Ontology (GO) Enrichment and Pathway Analysis of the Differentially Expressed Proteins

**Figure 2:**
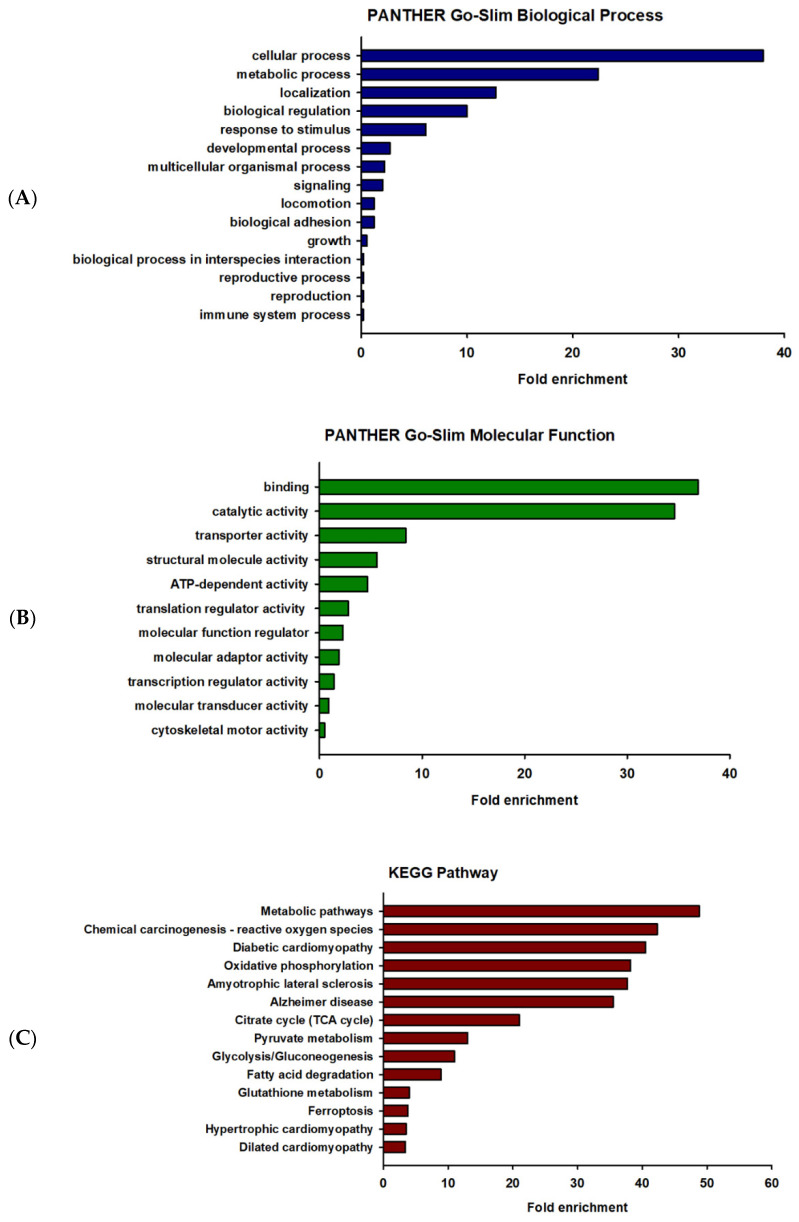
Gene ontology enrichment analysis of differentially expressed proteins in prometryn-treated mice heart. (**A**) The hub genes expressed in terms of biological process. (**B**) The hub genes expressed in terms of molecular function. (**C**) KEGG pathway enrichment analysis. Significantly enriched GO enrichment analysis was performed using PANTHER classification system and KEGG pathway based on the number of differentially expressed genes.

GO enrichment analysis is widely used to identify the biological roles and functions of a specific gene and its products. All differentially expressed proteins were mapped to their enriched GO terms based on the functional annotation using the PANTHER database. The differentially expressed proteins were categorized into two classes, including biological process and molecular function, as shown in Figure 2. In terms of biological process, the majority of the protein class that were altered were associated with cellular process (GO:0009987) (156 proteins) and metabolic process (GO:0008152) (92 proteins), as represented in Figure 2A. The proteins of molecular function were mainly involved in binding (GO: 0005488) (79 proteins) and catalytic activity (GO:0003824) (74 proteins), as represented in Figure 2B. To identify the enriched pathways among the differentially expressed proteins, KEGG pathway analysis was performed using the online software g:Profiler (http://biit.cs.ut.ee/gprofiler/gost, accessed on 20 October 2022). The results of the KEGG pathway analysis were ranked by enrichment score and the pathways with *p*-value < 0.001. The enriched pathways in the differentially expressed proteins of vehicle-treated and prometryn-treated male mice heart include, but not limited to, metabolic pathways (KEGG:01100), chemical carcinogenesis—reactive oxygen species (KEGG:05208), and diabetic cardiomyopathy (KEGG:05415) (Figure 2C). Other proteins that were differentially expressed as well as a heatmap of some of the differentially expressed proteins are shown in Supplementary Figures S1–S5. A detailed list of proteins detected and quantification levels are shown in Supplementary Table S1.

### 2.4. Prometryn Increased Antioxidant Defense System Proteins

**Figure 3:**
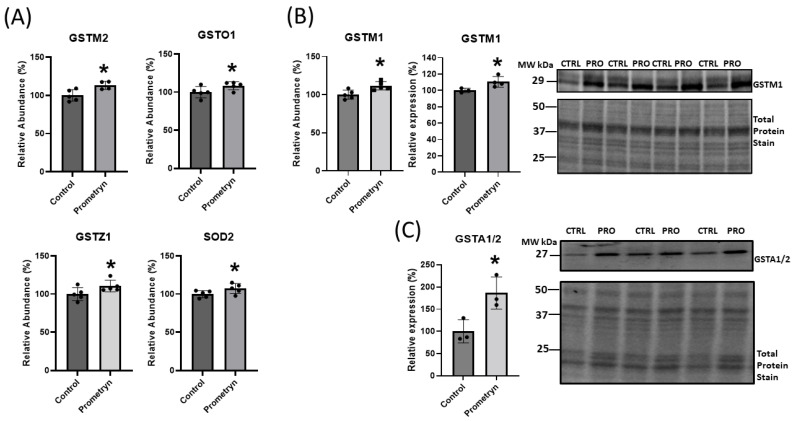
Short-term prometryn treatment induced oxidative stress-related proteins in mice heart. (**A**) Increased abundance of proteins associated with detoxification of oxidative stress: glutathione S-transferase Mu (GSTM2), glutathione S-transferase Omega 1 (GSTO1), glutathione S-transferase Zeta 1 (GSTZ1), and mitochondrial superoxide dismutase 2 (SOD2) obtained via mass spectrometry. (**B**) Increased abundance of glutathione S-transferase Mu 1 (GSTM1) obtained with mass spectrometry and independent validation using immunoblot analysis in mouse heart. (**C**) Prometryn treatment significantly increased the protein expression of glutathione S-transferase alpha 1/2 (GSTA1/2) in mouse heart. Band signal intensities were analyzed using Image Lab^®^ Software Version 12 (Bio-Rad, Hercules, CA, USA) and normalized with intensities obtained from loading control after staining with commercial Ponceau S for total protein. Values are mean ± SD; *n* = 3–5 per group. * *p* < 0.05.

There is a steady-state balance between ROS and cellular antioxidant systems during normoxia. However, the overproduction of ROS in intra- or extracellular spaces can occur due to environmental factors, such as exposure to pesticides, which can result in cellular dysfunction and apoptosis [1]. Our proteomic analysis showed that various antioxidant enzymes such as glutathione S-transferase Mu 1 (GSTM1), glutathione S-transferase Mu 2 (GSTM2), glutathione S-transferase Omega 1 (GSTO1), glutathione S-transferase Zeta 1 (GSTZ1), and mitochondrial superoxide dismutase 2 (SOD2) were upregulated in prometryn-treated male mice hearts in comparison to vehicle-treated male mice hearts (Figure 3A). Additionally, we independently validated the proteomics results by analyzing the protein expression levels of GSTM1 and glutathione S-transferase alpha 1/2 (GSTA1/2) via immunoblotting. The expression levels of GSTM1 and GSTA1/2 were significantly upregulated between prometryn-treated male hearts, suggesting that the immunoblotting data were consistent with the proteomics data (Figure 3B,C). Together, our data suggest that prometryn-treated male mice hearts were under oxidative stress conditions due to the observed elevated antioxidant enzymes that are involved in the detoxification and metabolism of xenobiotics and carcinogens and response to oxidative stress.

### 2.5. Prometryn Treatment Induced Proteasomal Dysfunction

**Figure 4:**
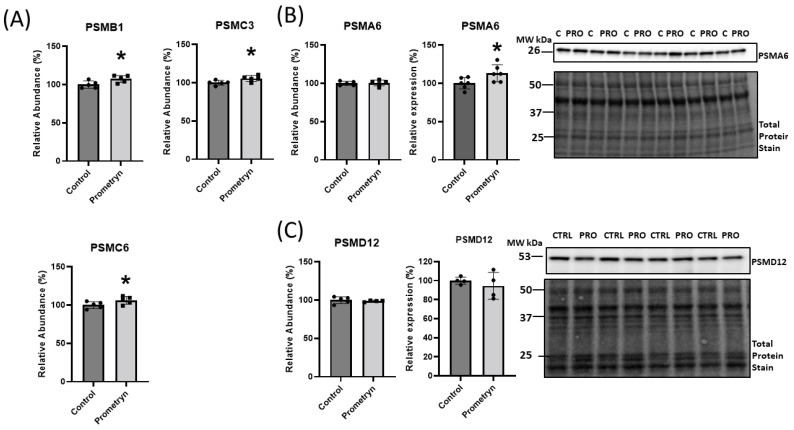
Effects of prometryn treatment on 26S proteasome expression levels in mice heart. (**A**) Changes in the abundance level of proteasome 20S subunit beta 1 (PSMB1), proteasome 26S subunit, ATPase (PSMC3), and proteasome 26S regulatory subunit 10B (PSMC6) obtained via mass spectrometry. (**B**) Proteasome 20S subunit alpha 6 (PSMA6) abundance level showed minimal change from obtained mass spectrometry data. However, PSMA6 protein expression showed significant increase in prometryn-treated mouse heart validated via immunoblotting. (**C**) Proteasome 26S non-ATPase regulatory subunit 12 (PSMD12) abundance level and protein expression level both decreased in data obtained from mass spectrometry and immunoblotting, respectively. Band signal intensities were analyzed using Image Lab^®^ Software Version 12 and normalized with intensities obtained from loading control after staining with commercial Ponceau S for total protein. Values are mean ± SD; *n* = 4–6 per group. * *p* < 0.05.

**Figure 5:**
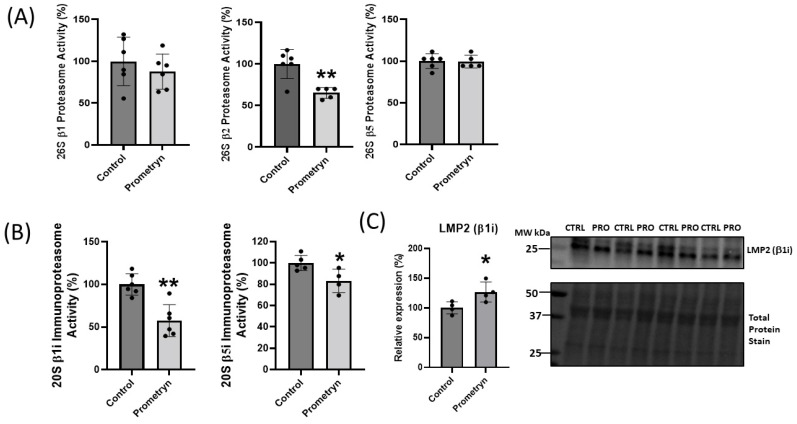
Prometryn treatment decreased 26S proteasome and 20S immunoproteasome activities in mice heart. (**A**) 26S proteasome (β1, β2 and β5) activities of heart lysates from male mice. (**B**) β1i and β5i immunoproteasome activities of male mice lysates. (**C**) Protein expression level of LMP2 (β1i) was increased in male mice heart from prometryn group. Values are mean ± SD; *n* = 4–6 per group. * *p* < 0.05, ** *p* < 0.001.

The proteomics data uncovered differential expression of a number of proteins associated with the ubiquitin-proteasome system (UPS), implicating a potential function of these proteins in oxidative stress responses. The UPS plays an essential role in the degradation of altered and misfolded proteins due to oxidation [17]. With prometryn treatment, the proteomics showed that the level of proteasome 20S subunit beta 1 (PSMB1), proteasome 26S subunit, ATPase (PSMC3), and proteasome 26S regulatory subunit 10B (PSMC6) were significantly upregulated in male mice heart. In contrast, proteasome 20S subunit alpha 6 (PSMA6) showed minimal change (Figure 4B). On the other hand, the data showed a decrease in the level of proteasome 26S non-ATPase regulatory subunit 12 (PSMD12) in prometryn-treated heart (Figure 4C). We then employed immunoblotting to further validate the proteomics data. The immunoblotting protein expression level of PSMD12 was decreased in prometryn-treated hearts, consistent with the proteomics data (Figure 4C). However, the protein expression level of PSMA6 was significantly increased (Figure 4B). It has been previously shown that a decline in proteasome activity might be associated with various pathological conditions such as aging, cardiomyopathies, heart failure, and chronic neurodegenerative diseases [18,19,20]. Therefore, we investigated the effects of prometryn treatment on the three independent proteasome catalytic subunit activities. We found that the β1 (caspase-like) and β2 (trypsin-like) proteasome activities were decreased in prometryn-treated heart. At the same time, there was no change in the β5 (chymotrypsin-like) proteasome activity (Figure 5A). Since there was a decline in proteasome subunit activity, we decided to measure immunoproteasome catalytic subunit activity. The constitutive proteasome β1, β2 and β5 subunits of 20S proteasome can be replaced by β1i, β2i, and β5i immunoproteasome counterparts during stress conditions, respectively. Our results suggest that both β1i and β5i immunoproteasome subunit activities were significantly decreased in prometryn-treated male mice hearts compared to control (Figure 5B). Lastly, we found that the protein expression level of LMP2 (β1i) was significantly elevated in prometryn-treated mice heart (Figure 5C).

### 2.6. Prometryn Affected Glycolytic Pathway in Male Mice Heart

**Figure 6:**
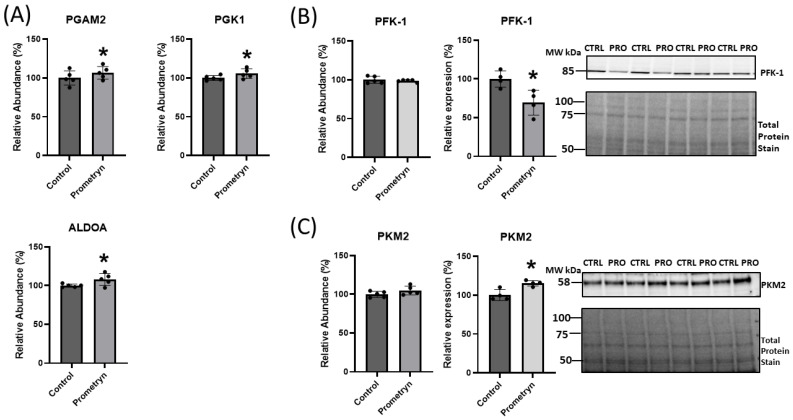
Proteomics uncovered differentially changes in proteins related to glycolytic pathway in prometryn-treated mice heart. (**A**) Changes in the abundance level of phosphoglycerate mutase 2 (PGAM2), phosphoglycerate kinase 1 (PGK1), and fructose-bisphosphate aldolase (ALDOA) obtained via mass spectrometry. (**B**) Phosphofructokinase-1 (PFK-1) protein abundance decreased in mass spectrometry data and validation via immunoblotting analysis showed significant decrease in PFK-1 protein expression level due to prometryn treatment. (**C**) Pyruvate kinase muscle isozyme 2 (PKM2) abundance level and protein expression level both increased in data obtained from mass spectrometry and immunoblotting, respectively. Band signal intensities were analyzed using Image Lab^®^ Software Version 12 and normalized with intensities obtained from loading control after staining with commercial Ponceau S for total protein. Values are mean ± SD; *n* = 4–5 per group. * *p* < 0.05.

Pathway analysis identified the glycolytic pathway as one of the enriched pathways among the differentially affected pathways in prometryn-treated hearts. The proteomic data revealed that several proteins related to the glycolytic pathway were either upregulated or downregulated in prometryn-treated male mice heart compared to control. There was a significant increase in the abundance of phosphoglycerate mutase 2 (PGAM2), phosphoglycerate kinase 1 (PGK1), and fructose-bisphosphate aldolase (ALDOA) in prometryn-treated heart when compared to the control group (Figure 6A). The mass spectrometry data also showed an increase in the abundance of pyruvate kinase muscle isozyme 2 (PKM2) and a decrease in the abundance of phosphofructokinase-1 (PFK-1) in the hearts of mice treated with prometryn when we compared it to the hearts of vehicle-treated mice (Figure 6B,C). Secondary validation of the expression levels of two proteins associated with glycolytic pathways was carried out using immunoblotting. The immunoblotting data showed that the protein expression level of PKM2 was significantly increased while that of PFK-1 was significantly decreased, consistent with the proteomics data (Figure 6B,C). Our data demonstrated that prometryn increased the levels of PGAM2, PGK1, ALDOA, and PKM2, while it decreased the level of PFK-1.

### 2.7. Mitochondrial Function Impaired in Prometryn-Treated Male Mice Heart

**Figure 7:**
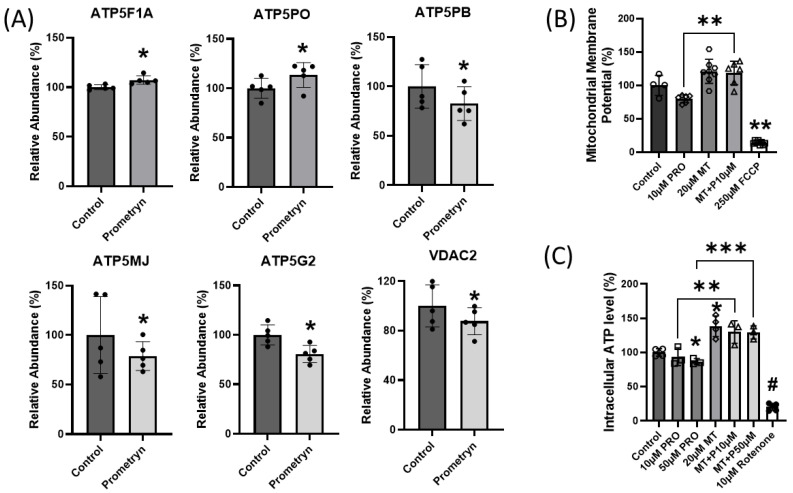
Prometryn treatment induced mitochondrial dysfunction in mice heart. (**A**) Changes in abundance level of ATP synthase F1 subunit alpha, mitochondrial (ATP5F1A), ATP syntha5Pse subunit O, mitochondrial (ATP5PO), ATP synthase F(0) complex subunit B1, mitochondrial (ATP5PB), ATP synthase membrane subunit J, mitochondrial (ATP5MJ), and ATP synthase, H+ transporting, mitochondrial F0 complex, subunit C2 (subunit 9) (ATP5G2) obtained via mass spectrometry. (**B**) Mitochondrial membrane potential was monitored using JC-10 in H9c2 cardiac cells. With prometryn treatment, mitochondrial membrane potential became more depolarized, relative to control. Meanwhile, MitoTEMPO reversed the effect of prometryn when added together. (**C**) Intracellular ATP levels in H9c2 cardiac cells were measured 24 h after treatment with prometryn. The luminescence intensity (L.I.) was normalized to the untreated cells level in each individual well. ATP levels were measured using firefly luciferin–luciferase luminescence assay. PRO = Prometryn, MT = MitoTEMPO, MT + P = MitoTEMPO + Prometryn, FCCP = Carbonyl cyanide p-(tri-fluoromethoxy)phenyl-hydrazone. Values are expressed as mean ± SD (*n* ≥ 3). * *p* < 0.05, ** *p* < 0.001, *** *p* < 0.0005, # represents **** *p* < 0.0001.

It is well established that the heart depends on a dense network of continuous mitochondrial ATP supply in order to develop force and maintain its continuous mechanical work [21,22]. Elevated stress is harmful to the constantly beating heart because it might affect different cell compositions of the heart [22]. Since the heart is densely packed with mitochondria, there is a need for proper communication with these mitochondria in order to sufficiently produce energy on a beat-to-beat basis [22,23]. Interestingly, the proteomics data uncovered significant changes in proteins involved in oxidative phosphorylation within mitochondria. ATP synthase F1 subunit alpha, mitochondrial (ATP5F1A) and ATP syntha5Pse subunit O, mitochondrial (ATP5PO) were significantly increased while ATP synthase F(0) complex subunit B1, mitochondrial (ATP5PB), ATP synthase membrane subunit J, mitochondrial (ATP5MJ), ATP synthase, H+ transporting, mitochondrial F0 complex, subunit C2 (subunit 9) (ATP5G2), and voltage-dependent anion channel 2 (VDAC2) were all significantly decreased in prometryn-treated male mice hearts compared to the control hearts (Figure 7A).

To determine the in vitro effects of prometryn treatment on mitochondrial function, we examined mitochondrial membrane potential and ATP level in H9c2 cardiac cells. After prometryn treatment for 24 h, the mitochondrial membrane potential was significantly depolarized (Figure 7B). However, when we pretreated the cells with 20 μM MitoTEMPO for 1 h before adding prometryn, there was a reversal in the depolarized mitochondrial membrane potential previously seen (Figure 7B). Controls with the addition of MitoTEMPO alone increased membrane potential relative to control cells, suggesting that mitochondrial superoxide ions are critical for heathy membrane potentials. Mitochondrial membrane potential is a key indicator of mitochondrial activity as it is related to the transmembrane potential of hydrogen ions which is harnessed to make ATP. The intracellular ATP level in H9c2 cardiac cells after 24 h treatment with prometryn was determined, and prometryn was found to significantly decrease ATP levels (Figure 7C). Addition of 20 μM MitoTEMPO prevented a prometryn-induced decrease in ATP level (Figure 7C). Taken together, the proteomic data demonstrated that prometryn altered the expression levels of several mitochondrial complex V proteins in male mice heart samples. Furthermore, biochemical assays measuring the mitochondrial membrane potential and ATP level shows that their values were decreased in H9c2 cardiac cells when treated with prometryn. These results indicate that mitochondrial function was significantly impaired, which may partly contribute to, or be the result of, an increased susceptibility to increased ROS generation/mitochondrial superoxide anion.

### 2.8. Prometryn Affects Fatty Acid Metabolism Pathway in Male Mice Heart

Gene ontology and pathway analysis from proteomic profiling identified that several biological processes involved in fatty acid beta-oxidation (GO:0006635), fatty acid metabolic process (GO:0006631), and fatty acid catabolic process (GO:0009062) were affected by prometryn treatment in mice (Table 1). Further analysis showed that prometryn treatment significantly altered at least 12 proteins associated with fatty acid metabolism. There were significant increases in electron transfer flavoprotein subunit beta (ETFB), hydroxyacyl-CoA dehydrogenase trifunctional multienzyme complex subunit alpha (HADHA), enoyl-CoA delta isomerase 2 (ECI2), 2,4-dienoyl-CoA reductase 1 (DECR1), acetyl-CoA acyltransferase 2 (ACAA2), Enoyl-CoA hydratase, short-chain 1 (ECHS1), 17-β-hydroxysteroid dehydrogenase type 10 (HSD17B10), and fatty acid-binding protein 5 (FABP5) in prometryn-treated heart when compared to the control group. Although most of the proteins related to fatty acid metabolism were increased, there was also a significant decrease in the abundance level of some proteins associated with fatty acid metabolism, such as carnitine palmitoyltransferase 1B (CPT1B), electron transfer flavoprotein dehydrogenase (ETFDH), ATP-binding cassette subfamily D member 3 (ABCD3), and solute carrier family 25 member 20 (SLC25A20) (Supplementary Figure S2).

### 2.9. Prometryn Affects Calcium Signaling Pathways

While the pathway analysis did not identify calcium signaling pathways as one of the major pathways affected by prometryn, manual investigation of the results showed that two enzymes critical for calcium signaling, the sarcoplasmic/endoplasmic reticulum calcium ATPase 2 (ATP2A2) and ryanodine receptor 2 (RYR2), were both decreased in the hearts of prometryn-treated mice (Supplementary Table S1). Two other proteins, Aralar 1 and Aralar 2, calcium-binding mitochondrial carrier proteins, important in maintaining mitochondrial calcium levels were also decreased by prometryn (Supplementary Table S1).

## 3. Discussion

Previous evidence has established that some classes of pesticides, such as organochlorines and organophosphate, are deleteriously toxic, and long-term accidental exposure to these pesticides can result in adverse side effects such as gastrointestinal-, cardiac-, and hepato-toxicity, neurodegenerative diseases, cancers, reproductive disorder, and birth defects [1,24]. The majority of previous studies have examined the effects, impact, and toxicity of prometryn on soil, plant seedlings, and aquatic fishes [25,26,27,28]. However, there is currently little or no information available in the literature regarding the toxicity of prometryn to the heart and other organs involved in the circulatory system. Since prometryn is widely used across the world as an herbicide to control the growth of annual broadleaf and weeds in many cultivated plants, there are possible cases that humans are at risk of being exposed to prometryn and other commonly used pesticides more than expected. Prometryn is relatively persistent in waters, soil, and even air near application sites [29]. Significant traces of prometryn have been documented to be remnant in plants used for human and domestic animal consumption [30,31] and some medicinal plants that were protected with prometryn [32]. Prometryn can be found in trace amounts in rat, cow and human milk after the mother might have been in contact with prometryn one way or the other without direct exposure [33,34,35].

Interestingly, a study by Liu et al. showed that prometryn increased intracellular ROS in human bronchial epithelial BEAS-2B cell line [5]. However, the increased oxidative stress generation was only observed using 200 μM prometryn, which may be highly unlikely for humans to be directly exposed to prometryn at such a high concentration. They also found that prometryn induced DNA double-strand breaks (DNA damage) in BEAS-2B cells [5]. Another study by Boulahia et al. reported that prometryn induced a significant oxidative stress response in common bean (*Phaseolus vulgaris* L. Seedlings) [25]. The authors found that soil treated with prometryn at the highest doses of 100 and 500 μM prometryn led to lipid peroxidation (an indicator of oxidative stress), affected antioxidant activities, and affected the growth parameters of the plant [25]. However, at a low concentration of 10 μM prometryn, they observed increased antioxidant enzymatic activities without affecting growth or lipid peroxidation [25]. In our study, prometryn had harmful effects in cardiac cells even at low concentrations. We observed increased ROS and mitochondrial damage, decreased ATP level, and decreased cell viability at 10–30 μM prometryn in cardiac cells. The in vitro experiment results from our study suggest that the heart might be more sensitive to prometryn exposure than previously investigated cells or plants. Previous research suggests that prometryn acts as an inhibitor of chloroplast electron transfer chain (ETC) [36]. We found that the increased superoxide ions were responsible for the mitochondria dysfunction since using a mitochondrial ROS scavenger MitoTEMPO in the prometryn-treated groups prevented the deleterious effects of prometryn in H9c2 cardiac cells.

We sought to unravel molecular mechanisms and signaling pathways that could be impacted due to prometryn effect in the heart and circulatory system in whole. Only two publications that utilized mice/rats to study prometryn were found in the literature. These publications used leukocytes, thymus, spleen, and lymph nodes, and were published over 10 years ago [29,37]. We used 185 mg/kg body weight prometryn in our study based on the two previous publications that used mice. In the previous publications, three concentrations of prometryn were used, which were classified as low, medium, and high. The medium dose was 375 mg/kg which represents 1/10 LD_50_ for mouse, while 185 mg/kg and 555mg/kg was a twofold decrease or increase from that standard, respectively [29,37]. The authors found that prometryn at all concentrations induced DNA damage and fragmentation in leukocytes of mice sub-chronically exposed for 14 and 28 days [29]. They also concluded that prometryn or its metabolic residues that form during the break down of the compound (since it’s bio-transformed after absorption in the stomach) might have the potential to induce processes that causes genotoxic effects on mice leukocytes. In our studies, the mice were exposed to prometryn for a shorter ti1010me period, 7 days.

We hypothesized that exposure to prometryn would induce oxidative stress by disrupting mitochondrial function, thereby dysregulating an array of cardiac signaling pathways which eventually would lead to upregulation in apoptosis in the heart. In order to validate our hypothesis, we investigated the effects of prometryn in vivo using 10-week- old male mice. This is the first study involving identification and validation of proteins changed by prometryn in mice heart. In the in vivo model, we performed proteomics using LC-MS/MS which provides an unbiased insight into the changes and alterations at the proteome level. We found that prometryn affects major cellular pathways, such as the antioxidant defense system, ubiquitin-proteasome system, glycolysis, ATP synthesis and mitochondrial protein, TCA cycle, fatty acid metabolism and beta oxidation, and heat shock-related protein.

### 3.1. Proteins Involved in Antioxidant Defense System

Based on the mass spectrometry proteomics data, we uncovered upregulation of various antioxidant enzymes such as GSTM1, GSTM2, GSTO1, GSTZ1, PRDX6, and SOD2 in response to prometryn treatment in male mice heart. We also confirmed a significant upregulation in GSTA1/2 in prometryn-treated heart when compared to the control via immunoblotting. The primary function of these enzymes involves detoxification and elimination of xenobiotic compounds and toxic metabolites. GSTs are important detoxifying enzymes that are specifically able to conjugate glutathione (γ-l-glutamyl-l-cysteinyl-glycine, GSH) to a wide range of hydrophobic and electrophilic molecules and therefore play a critical role in cellular detoxification [38]. They play important roles in biotransformation of xenobiotics, glutathionylation, deglutathionylation, and redox detoxification [39]. Tiwari et al. reported that GSTM1 and GSTA1/2 were upregulated in livers of mice treated with medium-dose of ibuprofen for 7 days [40]. It has been reported that prometryn upregulated GST activity in Phaseolus vulgaris L. seedlings [25] and altered antioxidant enzyme activity in fish [26,27,28]. Jin et al. demonstrated that GSTM2 was highly up-regulated in hepatic steatosis tissues and high-fat diet-fed mice [41]. GSTO1 overexpression and polymorphism has been associated with several pathological diseases including neurological disorders, cancers, and inflammatory diseases. GSTO1 has been reported to be upregulated in several human cancers including esophageal squamous cell carcinoma, colorectal, and urinary bladder cancer [39]. Two other important antioxidant enzymes that were upregulated are peroxiredoxin-6 (PRDX6) and manganese superoxide dismutase (SOD2). PRDX6 plays a crucial role in protecting cells and tissues from oxidative damage, particularly by scavenging hydrogen peroxide and reducing lipid peroxides [42]. SOD2 is a critical enzyme that helps to protect cells and tissues from the damaging effects of oxidative stress by neutralizing superoxide radicals. Its presence in the mitochondria is especially important for safeguarding cellular energy production and preventing mitochondrial dysfunction [42]. SOD2 plays a role in maintaining redox balance which is essential for overall health and well-being.

Several heat shock proteins associated with the antioxidant defense system were also upregulated by prometryn, including heat shock protein (HSP) 60, HSP70, HSP75 and HSPB1. HSP60 is mainly localized in the mitochondria, while HSP70 is abundant in the cytoplasm but also found in many cellular organelles including the nucleus [43,44]. HSP75 has been associated with the protection of mitochondria against oxidative stress, while HSPB1 is associated with protection against oxidative stress [43]. These heat shock proteins help cells to cope with oxidative stress by assisting in the repair and removal of damaged proteins, stabilizing cellular structures, and indirectly regulating antioxidant enzymes. Their functions contribute to the overall protection of cells from the harmful effects of ROS and oxidative damage. The increase in antioxidant enzymes and HSPs related to oxidative stress most likely represents a protective response by the animal to counteract the harmful effects of oxidative stress induced by prometryn.

### 3.2. Proteins Involved in Degradation

The proteasome is a component of the ubiquitin-proteasome system (UPS), which is critical for protein quality control and is the main intracellular degradation pathway that eliminates polyubiquitinated, misfolded, and oxidized proteins by a multi-complex 26S proteasome [45]. The 26S proteasome mainly degrades polyubiquitinated proteins in an ATP-dependent manner and contains caspase-like, trypsin-like and chymotrypsin-like proteolytically active sites in the β1 (PSMB6), β2 (PSMB7), and β5 (PSMB5) subunits of the 20S core [45,46]. The subunits mediate cleavage after acidic (β1), basic (β2), or hydrophobic (β5) amino acids [46]. Mass spectrometry showed that proteins associated with proteasome complex were differentially regulated in the heart of male mice treated with prometryn. We found that PSMB1, PSMC3, and PSMC6 were significantly upregulated, while PSMA6 increased, but PSMD12 was downregulated in prometryn group when compared to the control group. This may suggest that 20S core subunits of the proteasome is upregulated, while the 26S cap is downregulated. The free 20S core is capable of degrading damaged and partly broken down proteins in an ATP independent manner. Although there are currently no studies that implicate prometryn in causing proteasome dysfunction, several other studies have showed that some pesticides are capable of affecting the ubiquitin-proteasome system [47,48,49,50]. Kiyosawa et al. showed that o,p′-dichlorodiphenyltrichloroethane (DDT), an agricultural pesticide and malarial vector control agent, significantly increased gene expression related to protein synthesis/degradation via proteasome. They found that o,p′-DDT induced differential gene expression of *Psma4*, *Psma5*, *Psma6*, *Psmb1*, *Psmb4*, *Psmb5*, *Psmc3*, and *Psmc5* in the liver of immature (20-day old) ovariectomized rats [51]. They concluded that the induction of proteolysis genes occurred in order to rapidly facilitate protein turnover and cellular adaptation to their observed hepatocyte swelling with hypertrophy and induction of genes associated with oxidative stress [51]. Our data suggest that prometryn might be acting the same as o,p′-DDT since we found increased abundance of proteolysis enzymes in our study. However, when we measured the activities of subunits of the 20S core of the 26S proteasome, we found that the β1 (PSMB6) and β2 (PSMB7) subunit activities were decreased while there was no change in β5 (PSMB5) subunit activity in prometryn-treated mice heart when compared to the control. Pesticides such as rotenone, paraquat, ziram, diethyldithiocarbamate, endosulfan, benomyl, dieldrin, carbendazim, cyanazine, ferbam, metam, propargite, and triflumizole have been shown to inhibit 26S proteasomal activity in different cells and animal models [48,49,50].

Upon interferon-γ (IFN-γ) induction, the constitutive β1, β2 and β5 subunits of 20S proteasome are incorporated into nascent catalytically active inducible β1i (LMP2), β2i (MECL1) and β5i (LMP7) subunits that are also referred as the immunoproteasome [40,52]. The immunoproteasome plays an important role in degrading oxidized proteins, preventing protein aggregation, cytokine production, antigen presentation and T cell differentiation, and survival [53,54]. Immunoproteasome is a proteasome variant that is found only in jawed vertebrates, and any alteration in the expression, assembly or function of the immunoproteasome can lead to cancer, autoimmune disorders, or inflammatory diseases [53]. We demonstrated that prometryn significantly decreased the β1i and β5i subunit activities in male mice heart. The amount of these subunits is lower in abundance compared to the constitutive subunits and as such are not often observed in proteomic experiments. The increased expression but lower proteolytic activities of the 20S proteasome suggest that upregulation of the proteasome may be as a response to decreased proteasome activity. It is possible that excess ROS produced from the exposure to prometryn is involved in this pathway, thereby decreasing the activity of the proteasome. A decreased immunoproteasome activity would result in a slower rate of removal of oxidized proteins which may partly account for some of the cardiotoxic effects observed in the cell viability assays. Altogether these results suggest that prometryn caused proteasome dysfunction in male mice heart.

Another pathway involved in intracellular protein degradation is the lysosomal system. Our data found that only one lysosomal proteolytic enzyme, cathepsin D (CTSD, Supplementary Table S1), was upregulated in the hearts of prometryn-treated mice. Cathepsin D is a key enzyme involved in the degradation of proteins within lysosomes and is important in cellular homeostasis [55]. These changes in the expression and activity of degradative enzymes will cause alterations in protein degradation which will have significant effects on heart function as the heart is a highly dynamic and metabolically active organ that relies on efficient protein turnover to maintain its structure and contractile function.

### 3.3. Proteins Involved in Energy Metabolism

Mass spectrometry showed that proteins associated with metabolic processes were amongst the largest group of differentially expressed proteins due to prometryn treatment in male hearts relative to controls. MS and immunoblotting showed that one of the key enzymes involved in regulating glycolysis, PKM2, was upregulated in the prometryn group relative to the control group. PKM2, one of the four isoforms of pyruvate kinase, is a terminal enzyme in the glycolytic pathway that catalyzes the conversion of phosphoenolpyruvate to pyruvate and promotes metabolic flux into the activation pentose phosphate pathway (PPP) [39,56,57]. However, higher levels of PKM2 gene expression has been associated with poor prognosis in multiple myeloma patients [58], as well as in other diseases like diabetic nephropathy [57,59]. When PKM2 was highly expressed it shifted the glucose metabolism from mitochondrial respiration to lactate production in tumor cells [60]. Song et al. showed that a classic persistent organic pollutant, 3,3′4,4′,5-pentachlorobiphenyl (PCB126), increased ROS production and PKM2 expression via the activation of PKM2/STAT3/Snail1 cascades in human hepatocellular carcinoma cells [61]. In addition, Morales-Prieto and Abril found that 1,1-dichloro-2,2-bis(p-chlorophenyl)ethylene (p,p′-DDE), a derivative of DDT, significantly increased the activities of three key glycolytic enzymes in the liver of the p,p′-DDE-exposed mice [62]. They found that the activities of glyceraldehyde-3-phosphate dehydrogenase (GAPDH), lactate dehydrogenase (LDH), which aids in the conversion of pyruvate to lactate that regenerates NAD^+^ needed for glycolysis, and glucose 6-phosphate dehydrogenase (G6PDH) which is the key regulatory enzyme of the pentose phosphate pathway and provides reduced NADPH for biosynthetic reactions, including lipogenesis, and to maintain the redox status of the cell, were all upregulated in the liver of the p,p′-DDE-exposed mice [62]. We hypothesize that the Warburg effect, which is an increase in the rate of glucose uptake even in the presence of oxygen, might better explain the phenomenon behind the observed upregulation of PKM2, PGAM2, PGK1, and ALDOA in prometryn-treated male mice hearts in our study.

Additionally, we observed a significant decrease in the expression level of PFK-1 in our immunoblotting data. PFK catalyzes the rate-limiting phosphorylation of fructose-6-phosphate and ATP to fructose-1,6-diphosphate and ADP [40]. Mice deficient in PFKM (*Pfkm^−/−^*) had high lethality around weaning, reduced lifespan, exercise intolerance and had progressive cardiac hypertrophy with age because of metabolic alterations [63]. Zanella and Bianchi demonstrated that pyruvate kinase deficiency leads to ATP depletion in RBCs, ultimately affecting the viability of the cells as well as accumulation of the glycolytic intermediates, 2,3-diphosphoglycerate (2,3-DPG) that eventually leads to impaired glycolytic flux through the inhibition of hexokinase [64]. This was consistent with our observed reduction in intracellular ATP levels in H9c2 cells. Notably, shortage in cellular ATP has been suggested to cause several diseases [65]. Moreover, impaired bioenergetics was shown to affect the redox balance of neurons [66]. ROS is capable of inducing bioenergetic changes in cells and tissues, such as in the liver where ROS induced changes in glucose and lipid metabolism [67]. Taken altogether, our results suggest that the observed changes in glycolytic enzymes might have arose as an adaptive response to compensate for the depletion of ATP against oxidative stress.

Since mitochondria produce over 90% of the ATP required by the high energy demands of cardiac tissues, mitochondrial disorders have been associated with the development of many cardiovascular diseases, including atherosclerosis, ischemic heart disease, cardiac hypertrophy, and heart failure [68]. Mass spectrometry unraveled differentially regulation of mitochondrial ATP synthases in our study. Mitochondrial ATP synthases are responsible for catalyzing ATP synthesis, utilizing an electrochemical gradient of protons across the inner membrane during oxidative phosphorylation [69]. We found upregulation of ATP5F1A and ATP5PO, while downregulation of ATP5PB, ATP5MJ, and ATP5G2 was markedly found in prometryn-treated mice heart. Chen et al. showed that ATP5G2 decreased in AMPKα2 KO mice hearts during caloric restriction, and they also observed increased oxidative stress and apoptosis in the same mice hearts [70]. In a mouse model of Leigh syndrome caused by a deficiency in a component of complex I, the levels of ATP were observed to dictate disease severity and neuronal death [71]. Consistently, Min et al. showed that 20 mg/L prometryn significantly increased ROS production level which resulted in decreased basal mitochondrial respiration and ATP production in zebrafish embryos after 96 h treatment. As a result, the group also found that the mRNA levels of genes associated with mitochondrial subunits I, III, and V were significantly reduced in zebrafish embryos due to exposure to prometryn [72]. In our data, we also observed reduced mitochondrial membrane potential, which indicates that prometryn is affecting oxidative phosphorylation and ATP synthesis. Prometryn typically acts by binding to the photosystem II D1 protein, which inhibits the plant cell photosynthetic electron chain. Photosynthetic electron transport chains have many similarities to other electron transport chains [5,73]. Therefore, we speculate that prometryn might induce oxidative stress in human and animal cells in a similar manner, thereby causing differential changes in proteins involved in energy metabolism. Another possible mechanism by which prometryn toxicity could be acting on cardiomyocytes is from the observed reduced intracellular ATP level which may result in alterations of K^+^ flux. Since cardiomyocytes possess ATP-sensitive K^+^ channels and are sensitive to oxidative stress, elevated levels of ROS production and impaired mitochondrial energetics may trigger K^+^ dispersion and a subsequent fall in the cardiomyocyte action potential, allowing the onset of heart conduction defects [12].

### 3.4. Proteins Involved in Fatty Acid Metabolism Pathway

There is a persistent high demand for energy to sustain the contractile activity of the heart due to the constant pumping of blood from the heart to the rest of the body. This demand is primarily carried out by the beta-oxidation of long-chain fatty acids to produce ATP [74]. It is estimated that about 70–90% of cardiac ATP comes from fatty acids, and the remaining 10–30% of cardiac ATP is derived from the oxidation of glucose, lactate, ketone bodies, and certain amino acids [75]. Therefore, it is important to understand the influence of exogenous compounds on cardiac ATP production and normal heart function. Proteomic profiling in this present study revealed that prometryn altered many enzymes involved in fatty acid metabolism. Interestingly, a previous study showed that two organochlorine pesticides; p,p′-dichlorodiphenyldichloroethylene (p,p′-DDE) and β-hexachlorocyclohexane (β-HCH), impaired mitochondrial function in mice liver via decreased fatty acid β-oxidation and aggravated disorders of fatty acid metabolism [76]. The authors found that β-HCH significantly lowered the mRNA expression of key enzymes for mitochondrial fatty acid β-oxidation. Particularly, the mRNA expression of carnitine palmitoyltransferase 1α (Cpt1α) was significantly decreased in their study [76]. Similarly, our proteomic data also showed that prometryn significantly decreased the abundance of Cpt1b (a muscular isoform of Cpt1α). Furthermore, a study by Yuan at al. found that down-regulation of SLC25A20 promotes hepatocellular carcinoma growth and metastasis through the suppression of fatty acid oxidation. We found that prometryn decreased the level of SLC25A20 in mice heart. Several clinical studies have also reported that deficiency and/or mutation in SLC25A20 resulted in neonatal hypoglycemia, arrhythmia and sudden death [77,78,79]. A study by Kankuri et al. showed that mice with left anterior descending coronary artery ligation-induced myocardial infarction (MI) and acute ischemic heart failure had decreased expression of SLC25A20 in their heart [80].

Another protein in the fatty acid metabolism pathway that was differentially expressed was ETFB. ETFB exist in the mitochondrial matrix and shuttles electrons between primary flavoprotein dehydrogenases. Zhao et al. discovered that the gene and protein expression levels of ATP5B and ETFB were upregulated in patients with hydronephrosis. Their results suggested that increasing levels of ATP5B and ETFB were associated with worsening renal injury [81]. Both of these proteins were upregulated in our study as well. Boudina et al. found that diabetic *db/db* mice hearts showed increased expression of ETFB as well as increased expression of HADHA and other proteins involved in fatty acid oxidation and electron transfer [82]. Our data also showed a significant increase in the level of HADHA by prometryn. Collectively, the data indicate that alterations in fatty acid β-oxidation represent a significant pathway through which prometryn modifies cardiac function. This is due to the pivotal role of fatty acid metabolism in cardiac performance, as it directly impacts the heart’s energy supply.

### 3.5. Other Pathway Proteins

In muscle tissue, contractile and calcium signaling pathways are often affected by xenobiotics. However, in this study, only myosin-binding protein C, cardiac-type (MYBPC3), was found to be significantly altered by prometryn. The increase in this protein in heart from prometryn-treated mice can have a range of effects on muscle function, including enhanced cardiac contractility and relaxation, and improved cardiac function [83].

In this proteomic investigation, we observed a noteworthy reduction in the abundance of ryanodine receptor 2 (RyR2) in the hearts of mice exposed to prometryn. Vatner et al. also found that there was a significant decrease in myocardial RyR receptors as well as altered excitation-contraction coupling in a canine model with pacing-induced congestive heart failure [84]. Since RyRs are proximally close to mitochondria, calcium (Ca^2+^) release from RyR has been shown to regulate ATP production in heart and pancreas cells [85,86,87]. This provides some insight into the potential mechanism of prometryn cardiotoxicity. Bround et al. reported that in vivo deletion of cardiac RyR2 in mice (cRyr2KO mice) reduced mitochondrial Ca^2+^, ATP levels, and a cascade of transcription factors involved in oxidative metabolism [85]. Hence, one possible mechanism by which prometryn might be acting is via reducing RyR2 levels leading to lower Ca^2+^ and NO release, decreasing ATP level, and contributing to impaired cardiac excitation–contraction coupling. Another important Ca^2+^ signaling protein that was downregulated in hearts by prometryn was the sarco/endoplasmic reticulum calcium ATPase (SERCA). After contraction, muscles need to relax, and Ca^2+^ is actively pumped back into the SR by SERCA [88]. A decrease in SERCA activity will impair the ability of the SR to reuptake Ca^2+^ from the cytoplasm. As a result, cytoplasmic Ca^2+^ levels will remain elevated, and muscle relaxation will be compromised. Decreased RyR and SERCA function will likely lead to dysregulation of intracellular Ca^2+^, which is critical for various cellular processes beyond muscle contraction. However, a more in-depth investigation is necessary to elucidate whether prometryn’s impact on the heart involves the inhibition of RyR2 and SERCA mechanisms.

Vascular endothelial cells line the entire circulatory system, from the heart to the smallest capillaries. Research has shown that the dysfunction of the vascular endothelium is directly involved in peripheral vascular disease, stroke, heart disease, diabetes, insulin resistance, chronic kidney failure, tumor growth, metastasis, venous thrombosis, and severe viral infectious diseases since the endothelial cells play an important role in regulating blood flow [89]. The endothelium performs its functions via specific junctional proteins and receptors that govern cell–cell and cell–matrix interactions as well as though the presence of membrane-bound receptors for numerous molecules including proteins, lipid-transporting particles, metabolites, and hormones [89,90]. Pathway analysis via gene ontology and proteomic profiling unraveled that six endothelial cell-related proteins, junction plakoglobin (JUP), desmoplakin (DSP), plakophilin 2 (PKP2), and fibrinogen family members (FGA, FGB, and FGG), were differentially expressed in the heart of mice treated with prometryn. A study by Wang et al. found that spiromesifen, which is a specific contact pesticide that has been extensively used to control the growth of sucking insects like mites and whiteflies on crops, triggered abnormal blood vessel development, including vascular deletions and malformations in zebrafish model. They also found that spiromesifen inhibited the proliferation and migration of vascular endothelial cells, thereby resulting in vascular malformation in vitro [91]. One of the mechanisms that is involved in the development of endothelial dysfunction in CVD is centered around the reduction in nitric oxide (NO) bioavailability. NO exerts diverse physiological actions, including vasodilation, anti-inflammation, antiplatelet, antiproliferation and antimigration [92]. Ghosh et al. found that four pesticides (β-endosulfan, aldrin, α-HCH and p,p’-DDE) increased the level of ROS which was accompanied by enhanced expression of NADPH oxidase and significantly decreased the level of NO in endothelial cells [93].

### 3.6. Other Potential Pathways

It should be noted that cellular signaling is complex, and other pathways are likely to be affected by prometryn that were not identified in this study. For example, proteomic profiling did not uncover any changes in the proteins involved in the guanylate cyclase system. However, it is possible that prometryn and other triazine pesticides with similar mechanisms of action might perturb the guanylate cyclase system. Nitric Oxide can stimulate the production of cGMP by interacting with the heme group of the soluble guanylate cyclase (sGC) [94]. Previous research has shown that some pesticides, such as paraquat, can affect the NO pathway, therefore affecting cGMP release [95]. Since sGC is the primary NO sensor and plays a central role in NO signaling, exogenous compounds such as prometryn might possibly affect the guanylate cyclase system. A significant reduction in NO could potentially lead to platelet aggregation and hypertension.

## 4. Materials and Methods

### 4.1. Cell Culture

H9c2 cardiac cells obtained from the ATTC (Manassas, VA, USA) were maintained in high glucose Dulbecco’s modified Eagle’s medium (DMEM, Gibco) supplemented with 2 mM L-glutamine, 110 mg/L sodium pyruvate, 100 U/mL penicillin, 100 μg/mL streptomycin and 10% fetal bovine serum (FBS). Cells were maintained in a humidified atmosphere containing 5% CO_2_ at 37 °C.

### 4.2. Cell Viability Assay

Cell viability was determined using alamarBlue (resazurin) reagent (Invitrogen, ThermoFisher Scientific, Eugene, OR) according to the manufacturer’s instructions. Briefly, cells were seeded into 96-well plate at a density of 1 × 10^4^ cells/well in a volume of 100 μL media and incubated overnight at 37 °C. The cells were treated with prometryn at different concentrations (10, 20, and 30 μM) or with the solvent alone (DMSO at a final concentration of 0.1%) or 200 μM H_2_O_2_ as positive control for 24 h in complete growth medium. After 24 h of treatment, the cells were washed twice with phosphate-buffered saline (PBS), and 10% (*v*/*v*) Alamar blue reagent was added to each well. Following 4 h incubation, the plate was placed on a microplate reader (TECAN Infinite M1000 Pro) and shaken for 1 min before the reading. Fluorescence was measured with excitation at 530 nm and emission at 590 nm.

### 4.3. Mitochondrial Membrane Potential

Mitochondrial membrane potential was evaluated using JC-10 (AdipoGen Life Sciences, San Diego, CA, USA). JC-10 is a superior alternative form of JC-1, a cyanine iodide dye. The cationic and lipophilic dye JC-10 readily accumulates in the mitochondrial matrix, where it forms red fluorescent aggregates in normal cells. Meanwhile, JC-10 diffuses out of the mitochondria and changes to its monomeric forms, and stains cells in green fluorescence in uncoupled, compromised cells [96]. Briefly, 1 × 10^4^ cells/well of H9c2 cells were seeded into a 96-well plate 24 h before the treatments. Cells were treated with prometryn for 24 h at different concentrations or pretreated 1 h ahead with 20 μM MitoTEMPO, a mitochondrial target antioxidant agent. Carbonyl cyanide p-(tri-fluoromethoxy)phenyl-hydrazone (FCCP) was used as a positive control. JC-10 (5 μM) was added to phenol-free medium, and cells were incubated for 1 h at 37 °C, 5% CO_2,_ and protected from light. The medium was removed, cells were washed once with PBS to remove background fluorescence, and 100 μL phenol-free media was added to the wells. The fluorescent intensities for aggregate and monomeric forms of JC-1 were measured at Ex/Em = 490/525 nm (FITC channel) and 540/595 nm (TRITC channel) with 100 μL phenol-free media, using a 96-well fluorometric plate reader (TECAN Infinite M1000 Pro). The aggregate/monomer ratio was proportional to the mitochondrial membrane potential.

### 4.4. Intracellular ATP Level Detection

Intracellular ATP was determined using a luminescent ATP detection assay kit (Abcam, Waltham, MA, USA). The quantitative determination of ATP was assessed using recombinant firefly luciferase and its substrate D-luciferin. The assay was based on luciferase’s requirement for ATP in producing light. To assess the intracellular ATP, H9c2 cells were seeded overnight in white-walled 96-well plate. H9c2 cells were treated with prometryn and MitoTEMPO for 24 h, and cell medium was removed afterwards. Then, detergent supplied in the kit was added and incubated for 5 min at room temperature. Substrate solution was added and incubated for 5 min at room temperature. Light emissions were acquired using a 96 microplate luminometer.

### 4.5. Intracellular Reactive Oxygen Species (ROS) Detection

The cell-permeant reagent 2′,7′–dichlorodihydrofluorescein diacetate (H2-DCFDA), a fluorogenic dye (Enzo Life Sciences, Inc., Farmingdale, NY, USA) was utilized to study the intracellular production of ROS in H9c2 cells. Briefly, 1 × 10^4^ cells/well of H9c2 cardiac cells were plated on a 96-well black plate and incubated overnight. The cells were washed twice with PBS, and then treated with 10 μM of H2DCFDA in phenol red free media for 30 min at 37 °C. Following incubation, the wells were washed again with PBS, and the cells were treated with different concentrations (10, 20, and 30 μM) of prometryn and 100 μM H_2_O_2_ (positive control) in phenol free media. ROS production was determined by measuring the fluorescence at Ex/Em at 502/523 nm.

CellROX Green (Invitrogen Detection Technologies, ThermoFisher Scientific, Carlsbad, CA, USA) was also used to additionally detect oxidative stress in H9c2 cardiac cells. CellROX Green Reagent is cell-permeable DNA dye that is non-fluorescent or very weakly fluorescent while in a reduced state. Upon oxidation, CellROX exhibit strong fluorogenic signal localized primarily in the nucleus and mitochondria. Briefly, 1 × 10^4^ cells/well of H9c2 cells were seeded on a 96-well black plate and incubated overnight. The cells were treated with 5 μM of CellROX Green for 30 min at 37 °C. Following incubation, the wells were washed three times with PBS, the cells were treated with 10–50 μM of prometryn, 100 μM H_2_O_2_ (positive control), or pretreated 1 h ahead with 10 μM MitoTEMPO, and then prometryn was added. ROS production was determined by measuring the fluorescence at Ex/Em at 485/520 nm.

### 4.6. Superoxide Anion Scavenging Assay

Superoxide generation was determined using the fluorescent probe MitoSOX Red mitochondrial superoxide indicator (Invitrogen Detection Technologies, ThermoFisher Scientific, Carlsbad, CA, USA). MitoSOX Red reagent is readily selective to the mitochondria, and inside the mitochondria, MitoSOX Red reagent can be oxidized by superoxide and it exhibits red fluorescence [15]. Briefly, H9c2 cells were seeded in a 96-well plate at a concentration of 1 × 10^4^ cells/well in 100 μL of complete medium and allowed to recover for 24 h. Cells were treated with prometryn at different concentrations for 24 h. Antimycin A was used as positive controls because it can induce an oxidative state in cells [15]. MitoSOX reagent 5 μM was added to the cells, and they were incubated for 30 min at 37 °C, 5% CO_2_ and protected from light. After staining with MitoSOX, the cells were washed once with 0.2% BSA in PBS to remove background fluorescence, and 100 μL/well of 0.2% BSA in PBS was added. The plate was read with a fluorescence microplate reader at Ex/Em at 510/580 nm.

### 4.7. Animal Studies

C57BL/6J male mice (10 weeks old) were used for the study. The animal experiment was performed in accordance with the protocols approved by the Institutional Animal Care and Use Committee (IACUC) of the University of California, Davis. This study is reported in accordance with the ARRIVE guidelines (https://arriveguidelines.org, accessed on 4 January 2021). Mice were maintained at controlled temperature and humidity and had free access to food and water. The control mice received pure corn oil subcutaneously every 48 h for 7 days. The prometryn treatment group mice were subcutaneously treated with 185 mg/kg of prometryn dissolved in pure corn oil every 48 h for 7 days. The dose of prometryn given to mice is approximately 1/20 of the LD_50 (mice)_ = 3750 mg/kg as previously described [29]. To avoid overdose through bioaccumulation (partition coefficient log pKo/w = 3.5), mice were given prometryn every 48 h for 7 days, prepared as a corn oil suspension at 100 μL per animal. None of the animals showed any clinical signs of disease or behavior disorders; at the end of the assay, all animals were alive. Their body weight was measured at the beginning and end of the experiment, and no differences were observed between the control and treated groups. The mice were euthanized using 1.5% isoflurane, and hearts were immediately excised and quickly washed twice in ice-cold PBS and then preserved in liquid nitrogen. Prior to use, the heart tissue was individually pulverized with a cryogenic-based metal tissue pulverizer and stored at −80 °C until use for proteomics and biochemical assays.

### 4.8. LC-MS/MS Using 10–Plex TMT Proteomics

Sample Preparation: Pulverized heart tissue (20 mg aliquot) was homogenized in lysis buffer (8 M urea, 50 mM TEAB (triethyl ammonium bicarbonate), pH 8) followed by sonication (three cycles for 10 s with 30 s wait in between on ice). The lysate was centrifuged at 15,000× *g* for 15 min at 4 °C, and the supernatant was separated. The protein concentration of the supernatant was measured using Bicinchoninic Acid (BCA) protein assay. A total of 100 µg (100 µL) of protein sample was removed and 5 µL of the 200 mM TCEP (tris(2-carboxyethyl) phosphine) was added and incubated at 55 °C for 1 h in a heat block. After that, 5 µL of 375 mM iodoacetamide was added to the sample and incubated for 30 min sheltered from light at room temperature. The samples were precipitated using 6 volumes (~600 µL) of pre-chilled (−20 °C) of acetone overnight. The next day samples were centrifuged at 10,000× *g* for 10 min at 4 °C, the supernatant was discarded, and the pellet was air dried.

Digestion of protein samples: The acetone-precipitated protein pellet was resuspended in 100 µL of 50 mM ammonium bicarbonate (NH_4_CO_3_). Briefly, 2.5 µL of trypsin (i.e., 2.5 µg) per 100 µg of protein was added to each sample and were incubated overnight at 37 °C. The digested samples were lyophilized with a speedvac vacuum concentrator and resuspended in 0.1% trifluoroacetic acid (TFA) and 5% acetonitrile.

TMT 10-plex peptide labeling: TMT label reagents were allowed to equilibrate to room temperature (RT) and anhydrous acetonitrile was added to each tube. TMT label reagents were added to each 20 µg of samples and were incubated for 1 h at RT. The reaction was quenched by adding 5% hydroxylamine to the labeled samples and incubated for 30 min at RT. All TMT labeled samples were combined in equal amounts in a new tube (200 µg total).

Fractionation: TMT-labeled samples were reconstituted in 0.1% TFA and the pH adjusted to 2. The combined sample (20 µg) was separated into 8 fractions using High pH Reverse-Phase Peptide Fractionation kit (ThermoFisher Scientific) with an extra wash before separation to remove extra labels. The eight fractions collected were dried almost to completion. 

LC-MS/MS: LC separation was carried out on a Dionex nano Ultimate 3000 (ThermoFisher Scientific) with a Thermo Easy-Spray source fitted with a PepSep emitter. The digested peptides were reconstituted in 2% acetonitrile/0.1% trifluoroacetic acid, and 5 µL of each sample was loaded onto a Thermo Scientific PepMap 100 C18 5 µm 0.3 × 5 mm reverse-phase trap where they were desalted online before being separated on a PepSep 25 cm × 150 µm × 1.5 µm reverse phase column. Peptides were eluted using a 90 min gradient of 0.1% formic acid (A) and 80% acetonitrile (B) with a flow rate of 500 nL/min. The separation gradient was ran using 3% to 5% B over 5 min, 5% to 27% B over 50 min, 27% to 55% B over 16 min, 55% B to 99% B over 4 min, a 2 min hold at 99% B, and finally 99% B to 2% B held at 2% B for 6 min.

MS3 Synchronous Precursor Selection Workflow: Mass spectra were acquired on a Fusion Lumos mass spectrometer (ThermoFisher Scientific) in a data-dependent MS3 synchronous precursor selection (SPS) method. MS1 spectra were acquired in the Orbitrap, 120K resolution, 50ms max inject time, and 5 × 10^5^ max inject time. MS2 spectra were acquired in the linear ion trap with a 1.2 Da isolation window, collision-induced dissociation (CID) fragmentation energy of 37%, turbo scan speed, 50 ms max inject time, 1 × 10^4^ automatic gain control (AGC), and with maximum parallelizable time turned on. MS2 ions were isolated in the ion trap and fragmented with a higher energy collisional dissociation (HCD) energy of 65%. MS3 spectra were acquired in the orbitrap with a resolution of 50K and a scan range of 100–500 Da, 105 ms max inject time, and 1 × 10^5^ AGC. 

Proteome Discoverer (PD) Search: Tandem mass spectra were processed and searched using Proteome Discoverer version 2.5. All spectra were recalibrated using the PD 2.5 recalibration node with the default parameters. MS3 reporter ions were quantified and detected using the most confident centroid with a mass tolerance of 20 ppm. MS/MS spectra were searched using SEQUEST-HT with trypsin enzyme specificity, max 2 missed cleavages, fragment ion tolerance of 0.6 Da, and precursor mass tolerance of 10 ppm. SEQUEST HT was set up to search the Mouse Uniprot proteome (UP000000589) and common laboratory contaminants from (http://thegpm.org.crap, accessed on 3 February 2022). Carbamidomethyl of cysteine, TMT10plex of lysine, and peptide N-termini were considered fixed modifications. Oxidation of methionine and acetyl of the protein N-terminus were specified in Sequest (XCorr Only) as variable modifications. SEQUEST HT results were processed with Percolator with a maximum Delta Cn of 0.05. Target decoy false discovery rates (FDR’s) were set at 1% with validation based on q-value.

Quantitative Data Analysis: Scaffold Q+ (version Scaffold 5.0.1, Proteome Software Inc., Portland, OR, USA) was used to quantitate Label Based Quantitation peptide and protein identifications. Peptide identifications were accepted if they could be established at greater than 96.0% probability of achieving an FDR less than 1.0% by the Peptide Prophet algorithm [97] with Scaffold delta-mass correction. Protein identifications were accepted if they contained at least 2 identified peptides. Protein probabilities were assigned via the Protein Prophet algorithm [98]. Proteins that contained similar peptides and could not be differentiated based on MS/MS analysis alone were grouped to satisfy the principles of parsimony. Normalization was performed iteratively (across samples and spectra) on intensities, as described in Statistical Analysis of Relative Labeled Mass Spectrometry Data from Complex Samples Using ANOVA [99]. Medians were used for averaging. Spectra data were log-transformed, pruned of those matched to multiple proteins, and weighted using an adaptive intensity weighting algorithm. Of 21,965 spectra in the experiment at the given thresholds, 15,251 (69%) were included in the quantitation. Differentially expressed proteins were determined by applying the Mann–Whitney Test with an unadjusted significance level of *p* < 0.05 corrected with Benjamini–Hochberg.

#### 4.8.1. 26S Proteasome Activity Assay

26S proteasome activity assay was carried out as described previously [100]. Briefly, 20 mg of pulverized heart samples were homogenized in 26S proteasome lysis buffer (50 mM Tris, 150 mM sodium chloride (NaCl), 1 mM ethylenediaminetetraacetic acid (EDTA), 5 mM magnesium chloride (MgCl_2_), 1 mM DTT (freshly added) [pH 7.5]) with a hand-held Potter-Elvehjem homogenizer. The homogenates were centrifuged at 12,000× *g* for 15 min at 4 °C, and the supernatant was collected. The protein concentration was quantified and diluted to 2 μg/μL concentration with the aforementioned lysis buffer. The AMC conjugated activity-specific peptides were used to assess the different proteasome catalytic subunit activities, β1 (caspase-like), β2 (trypsin-like), and β5 (chymotrypsin-like), and by adding 20 µg of protein diluted in 26S proteasome lysis buffer and 100 μM ATP. In order to control for non-proteasomal-mediated cleavage of substrates, a specific proteasome inhibitor, 10 μM bortezomib (β5 activity) and 100 μM bortezomib (β1 and β2 activities), was used in some wells, while dimethyl sulfoxide (DMSO) was used in wells without the proteasome inhibitor. Samples were incubated with inhibitors at RT for 20 min, protected from direct light exposure. Then, specific fluorescence-tagged AMC peptides for each proteasomal β subunits, 100 μM Z-LLE-AMC for β1, 100 μM Boc-LSTR-AMC for β2, and 100 μM Suc-LLVY-AMC for β5, were used to initiate the reaction. Proteasomal activity was assessed with continuous fluorescent measurement of the released AMC at 37 °C for 2 h using a fluorometric plate reader (Tecan Infinite M1000 Pro, Männedorf, Switzerland) at an excitation wavelength of 390 nm and an emission wavelength of 460 nm.

#### 4.8.2. Immunoproteasome Activity Assay

Immunoproteasome (β5i and β1i) activity assay was performed as previously described. Briefly, 20 mg of heart tissue samples were homogenized in immunoproteasome buffer (50 mM Tris, 5 mM MgCl_2_, 20 mM potassium chloride (KCl), 1 mM DTT (freshly added), pH 7.5) with a hand-held Potter-Elvehjem homogenizer. The homogenates were centrifuged at 12,000× *g* for 15 min at 4 °C and supernatant collected. Next, 20 μg of protein were incubated with immunoproteasome buffer, and specific immunoproteasome inhibitor or an equivalent volume of DMSO at RT for 20 min protected from direct light exposure. The specific inhibitors for β5i 20 µM ONX-0914 (Abmole Bioscience Inc., Houston, TX, USA, Cat. No. M2071) and β1i 50 µM bortezomib were used to control for non-immunoproteasome-mediated cleavage of substrates and evaluate the specificity of the assay. Then, 25 µM fluorogenic substrates Ac-ANW-2R110 (AAT Bioquest, Inc., Pleasanton, CA, USA) for β5i and Ac-PAL-2R110 (AAT Bioquest, Inc., Pleasanton, CA, USA) for β1i were used to initiate the reaction. The fluorescence intensity was measured every 5 min for 60 min at an excitation of 498 nm and an emission of 520 nm at 37 °C in a Tecan Infinite M1000 Pro fluorometer.

#### 4.8.3. Immunoblotting

##### Sample Preparation

Pulverized heart tissue (20 mg) was homogenized in either ice cold 1x RIPA buffer (50 mM Tris, 150 mM NaCl, 1% NP40, 0.5% sodium deoxycholate and 0.1% sodium dodecyl sulfate (SDS), pH 8) or lysis buffer (50 mM Tris, 150 mM NaCl, 1 mM EDTA, 5 mM MgCl2) [pH 7.5]) using a glass Dounce homogenizer. The homogenates were centrifuged at 12,000× *g* for 15 min at 4 °C, and the supernatant was collected. Protein concentrations were determined in triplicate using the BCA protein assay (Bio-Rad, Hercules, CA, USA, Cat. #500–0119) and were diluted to equal protein concentrations. Heart samples were mixed with 4X Laemmli sample buffer (8% SDS, 40% glycerol, 0.4% bromophenol blue, 240 mM Tris, pH 6.8) with freshly added β-mercaptoethanol, and the samples were denatured at 95 °C for 5 min.

##### Electrophoresis and Western Blotting

Equal amounts of protein (20 µg per lane) were separated on 4–20% TGX precast gels (Cat. # 567–1094, Bio-Rad) for 80 min at 120V. Proteins were transferred to a nitrocellulose membrane (Trans-Blot Turbo Midi Nitrocellulose, #170–4159, Bio-Rad) using the Trans-Blot Turbo Transfer System (Cat # 170–4155, Bio-Rad). The membranes were then stained with Ponceau S and imaged to serve as a loading control for total protein normalization of Western blots. Thereafter the membrane was blocked with 3% nonfat dry milk (NFM) (Cat. # 170–6404, Bio-Rad) in Tris-buffered saline (TBS) (50 mM Tris, 150 mM NaCl, pH 7.5) containing 0.05% (wt/vol) Tween 20 (TBST) at room temperature. The membranes were then incubated overnight at 4 °C with the following primary antibodies diluted in 1% TBST (see Table 2). The membranes were washed three times in TBST for 5 mins each. They were incubated at room temperature for 1 h with horseradish peroxidase-conjugated rabbit anti-mouse or anti-rabbit IgG secondary antibody (Sigma-Aldrich, anti-mouse Cat. # A9044, anti-rabbit Cat. # A0545) diluted 1:10,000 in 1% TBST. Blots were subsequently washed three times with 1X TBST for 5 mins each and developed using a commercial chemiluminescent reagent (Clarity, Bio-Rad-170–5061) and imaged using the ChemiDoc MP (Bio-Rad). All incubation steps carried out at 4 °C or RT were carried out with gentle shaking. The quantification of blots was conducted using Image Lab 5.0.

##### Statistical Analysis

For proteomics:

Differentially expressed proteins were determined by applying a Permutation Test with an unadjusted significance level of *p* < 0.05, which was then corrected using the Benjamini–Hochberg procedure to reduce the False Discovery Rate (FDR). After applying the Benjamini–Hochberg procedure, the corrected significance level was set at *p* < 0.00674.

For immunoblotting and biological assays:

Results are reported as mean ± standard deviation (SD), unless otherwise stated. Statistical significance was determined using a Student’s paired *t*-test when comparing two groups and one-way ANOVA when comparing multiple groups. A value of *p* < 0.05 was considered as statistically significant. Data analysis was carried out using GraphPad Prism 9.3.1 software (GraphPad Software, San Diego, CA, USA).

## 5. Conclusions

Persistent xenobiotics like prometryn, commonly found in the environment, can exert harmful effects on heart tissue. The cardiotoxicity associated with prometryn is likely to arise from its multifaceted impact on cardiac physiology. These effects encompass the generation of superoxides within mitochondria, resulting in mitochondrial dysfunction, impairment of proteasome and immunoproteasome activity, calcium signaling changes, and significant alterations in energy metabolism, thereby culminating in reduced ATP levels available for cardiac cells. The identification of key proteins and the elucidation of related signaling pathways that undergo modifications in response to prometryn exposure are fundamental in understanding the intricate molecular mechanisms behind its cardiac toxicity. Furthermore, these findings raise concerns regarding the potential development of cardiovascular diseases in individuals exposed to prometryn.

Expanding our perspective to encompass the broader implications of these discoveries within the context of human health is crucial. The adverse effects of persistent xenobiotics like prometryn on heart tissue underscore the importance of strict regulatory measures and proactive strategies to mitigate human exposure. Such measures should aim to safeguard public health and reduce the risk of cardiovascular disorders associated with environmental contaminants like prometryn. Exposures of prometryn at higher dosages and for longer durations are likely to cause more significant risks of diseases. In addition to exposure of the general population to prometryn in well water and some foods, farmers, agricultural workers, and individuals living near farms may be especially at risk due to frequent and prolonged contact with pesticides throughout the planting, growing, and harvesting seasons. It is essential for farmers and agricultural workers to receive proper training on pesticide safety, use appropriate prospective equipment, follow label instructions, and adopt best practices for handling and applying pesticides to minimize their risk of exposure. Overall, regulatory agencies need to continue to improve and enforce the guidelines and regulations in place to help protect the farmers and the environment from pesticide-related risks.

## Figures and Tables

**Table 1:** Biological processes in mice heart affected by prometryn treatment.

PANTHER GO-Slim Biological Process	Number of Differentially Expressed Proteins	Expected	+/−	Fold Enrichment	Raw *p* Value	False Discover Rate (FDR)
generation of precursor metabolites and energy (GO:0006091)	24	1.47	+	16.28	1.33 × 10^−20^	2.96 × 10^−17^
cellular respiration (GO:0045333)	19	0.8	+	23.81	3.36 × 10^−19^	3.73 × 10^−16^
energy derivation by oxidation of organic compounds (GO:0015980)	20	1.01	+	19.87	7.96 × 10^−19^	5.89 × 10^−16^
aerobic respiration (GO:0009060)	17	0.7	+	24.3	1.98 × 10^−17^	1.10 × 10^−14^
small molecule metabolic process (GO:0044281)	40	8.51	+	4.7	1.48 × 10^−15^	6.58 × 10^−13^
ATP metabolic process (GO:0046034)	15	0.83	+	17.97	7.11 × 10^−14^	2.63 × 10^−11^
ATP synthesis coupled proton transport (GO:0015986)	12	0.5	+	23.84	1.36 × 10^−12^	4.30 × 10^−10^
ATP biosynthetic process (GO:0006754)	12	0.52	+	23.28	1.73 × 10^−12^	4.26 × 10^−10^
electron transport chain (GO:0022900)	11	0.39	+	28.01	2.77 × 10^−12^	4.74 × 10^−10^
proton transmembrane transport (GO:1902600)	13	0.71	+	18.26	2.85 × 10^−12^	4.52 × 10^−10^
respiratory electron transport chain (GO:0022904)	10	0.38	+	26.28	4.68 × 10^−11^	4.16 × 10^−9^
oxidative phosphorylation (GO:0006119)	10	0.38	+	26.28	4.68 × 10^−11^	4.00 × 10^−9^
ribonucleotide biosynthetic process (GO:0009260)	14	1.2	+	11.64	8.83 × 10^−11^	7.00 × 10^−9^
protein folding (GO:0006457)	14	1.23	+	11.41	1.12 × 10^−10^	8.61 × 10^−9^
ATP synthesis coupled electron transport (GO:0042773)	8	0.28	+	28.34	2.61 × 10^−9^	1.49 × 10^−7^
organophosphate metabolic process (GO:0019637)	22	4.84	+	4.55	8.84 × 10^−9^	4.90 × 10^−7^
carboxylic acid metabolic process (GO:0019752)	20	4.05	+	4.94	1.18 × 10^−8^	6.41 × 10^−7^
organophosphate biosynthetic process (GO:0090407)	17	2.86	+	5.94	1.21 × 10^−8^	6.37 × 10^−7^
tricarboxylic acid cycle (GO:0006099)	7	0.23	+	30.02	1.93 × 10^−8^	9.72 × 10^−7^
mitochondrial ATP synthesis coupled electron transport (GO:0042775)	7	0.27	+	25.92	4.43 × 10^−8^	2.19 × 10^−6^
transmembrane transport (GO:0055085)	25	6.87	+	3.64	5.69 × 10^−8^	2.75 × 10^−6^
organic acid metabolic process (GO:0006082)	20	4.59	+	4.36	8.37 × 10^−8^	3.95 × 10^−6^
cellular amide metabolic process (GO:0043603)	21	5.17	+	4.06	1.20 × 10^−7^	5.56 × 10^−6^
ion transmembrane transport (GO:0034220)	21	5.31	+	3.95	1.88 × 10^−7^	8.51 × 10^−6^
peptide metabolic process (GO:0006518)	18	4.01	+	4.48	2.53 × 10^−7^	1.12 × 10^−5^
inorganic ion transmembrane transport (GO:0098660)	18	4.11	+	4.38	3.54 × 10^−7^	1.54 × 10^−5^
signaling (GO:0023052)	8	31.66	−	0.25	4.32 × 10^−7^	1.84 × 10^−5^
cell communication (GO:0007154)	8	31.72	−	0.25	4.37 × 10^−7^	1.83 × 10^−5^
signal transduction (GO:0007165)	7	29.5	−	0.24	5.52 × 10^−7^	2.27 × 10^−5^
aerobic electron transport chain (GO:0019646)	6	0.25	+	24.44	5.65 × 10^−7^	2.28 × 10^−5^
ion transport (GO:0006811)	24	7.44	+	3.23	8.46 × 10^−7^	3.35 × 10^−5^
carbohydrate derivative metabolic process (GO:1901135)	20	5.38	+	3.72	9.12 × 10^−7^	3.55 × 10^−5^
regulation of biological process (GO:0050789)	34	66.36	−	0.51	1.50 × 10^−6^	5.73 × 10^−5^
regulation of cellular process (GO:0050794)	32	63.53	−	0.5	2.11 × 10^−6^	7.92 × 10^−5^
cation transmembrane transport (GO:0098655)	17	4.21	+	4.04	2.15 × 10^−6^	7.95 × 10^−5^
cell surface receptor signaling pathway (GO:0007166)	0	13.22	−	<0.01	2.41 × 10^−6^	8.79 × 10^−5^
mitochondrion organization (GO:0007005)	11	1.73	+	6.36	2.60 × 10^−6^	9.32 × 10^−5^
inorganic cation transmembrane transport (GO:0098662)	16	3.81	+	4.2	2.62 × 10^−6^	9.25 × 10^−5^
carbohydrate derivative biosynthetic process (GO:1901137)	15	3.38	+	4.44	2.86 × 10^−6^	9.92 × 10^−5^
amide biosynthetic process (GO:0043604)	15	3.73	+	4.02	9.04 × 10^−6^	3.09 × 10^−4^
cation transport (GO:0006812)	18	5.27	+	3.42	9.85 × 10^−6^	3.31 × 10^−4^
biological regulation (GO:0065007)	41	71.93	−	0.57	1.08 × 10^−5^	3.58 × 10^−4^
inner mitochondrial membrane organization (GO:0007007)	4	0.15	+	27.16	3.43 × 10^−5^	1.02 × 10^−3^
nucleic acid metabolic process (GO:0090304)	12	32.07	−	0.37	3.79 × 10^−5^	1.11 × 10^−3^
peptide biosynthetic process (GO:0043043)	13	3.25	+	4	3.80 × 10^−5^	1.09 × 10^−3^
cellular modified amino acid metabolic process (GO:0006575)	7	0.86	+	8.15	4.16 × 10^−5^	1.18 × 10^−3^
cellular response to stimulus (GO:0051716)	15	35.84	−	0.42	5.89 × 10^−5^	1.65 × 10^−3^
mitochondrial membrane organization (GO:0007006)	4	0.2	+	20.37	8.79 × 10^−5^	2.30 × 10^−3^
immune system process (GO:0002376)	1	12.26	−	0.08	8.83 × 10^−5^	2.25 × 10^−3^
cellular component assembly (GO:0022607)	25	10.64	+	2.35	1.09 × 10^−4^	2.75 × 10^−3^
carboxylic acid catabolic process (GO:0046395)	7	1.06	+	6.63	1.39 × 10^−4^	3.47 × 10^−3^
mitochondrial electron transport, ubiquinol to cytochrome c (GO:0006122)	3	0.07	+	40.74	1.40 × 10^−4^	3.46 × 10^−3^
regulation of nucleobase-containing compound metabolic process (GO:0019219)	7	22.92	−	0.31	1.44 × 10^−4^	3.52 × 10^−3^
organic acid catabolic process (GO:0016054)	7	1.08	+	6.48	1.59 × 10^−4^	3.83 × 10^−3^
small molecule catabolic process (GO:0044282)	8	1.5	+	5.34	1.91 × 10^−4^	4.56 × 10^−3^
monocarboxylic acid catabolic process (GO:0072329)	5	0.49	+	10.18	2.08 × 10^−4^	4.90 × 10^−3^
organonitrogen compound metabolic process (GO:1901564)	55	33.61	+	1.64	2.76 × 10^−4^	6.38 × 10^−3^
transport (GO:0006810)	44	25.27	+	1.74	3.28 × 10^−4^	7.50 × 10^−3^
fatty acid beta-oxidation (GO:0006635)	4	0.29	+	13.58	3.44 × 10^−4^	7.80 × 10^−3^
mitochondrial electron transport, cytochrome c to oxygen (GO:0006123)	3	0.11	+	27.16	3.58 × 10^−4^	8.02 × 10^−3^
localization (GO:0051179)	52	31.53	+	1.65	3.88 × 10^−4^	8.62 × 10^−3^
regulation of nitrogen compound metabolic process (GO:0051171)	12	28.84	−	0.42	4.42 × 10^−4^	9.72 × 10^−3^
protein-containing complex subunit organization (GO:0043933)	15	5.41	+	2.77	4.88 × 10^−4^	1.06 × 10^−2^
phosphate-containing compound metabolic process (GO:0006796)	25	11.81	+	2.12	4.97 × 10^−4^	1.07 × 10^−2^
establishment of localization (GO:0051234)	44	25.65	+	1.72	5.22 × 10^−4^	1.11 × 10^−2^
phosphorus metabolic process (GO:0006793)	25	11.87	+	2.11	5.27 × 10^−4^	1.11 × 10^−2^
sulfur compound metabolic process (GO:0006790)	8	1.78	+	4.49	5.74 × 10^−4^	1.20 × 10^−2^
regulation of metabolic process (GO:0019222)	15	32.5	−	0.46	6.15 × 10^−4^	1.28 × 10^−2^
regulation of gene expression (GO:0010468)	10	25.2	−	0.4	6.55 × 10^−4^	1.35 × 10^−2^
regulation of macromolecule metabolic process (GO:0060255)	14	30.99	−	0.45	6.73 × 10^−4^	1.37 × 10^−2^
cellular process (GO:0009987)	156	127.69	+	1.22	7.18 × 10^−4^	1.45 × 10^−2^
regulation of primary metabolic process (GO:0080090)	13	29.21	−	0.45	7.20 × 10^−4^	1.44 × 10^−2^
response to stimulus (GO:0050896)	25	44.54	−	0.56	8.93 × 10^−4^	1.75 × 10^−2^
immune response (GO:0006955)	1	10.11	−	0.1	8.94 × 10^−4^	1.74 × 10^−2^
lipid oxidation (GO:0034440)	4	0.39	+	10.18	9.18 × 10^−4^	1.77 × 10^−2^
cellular component biogenesis (GO:0044085)	25	12.15	+	2.06	9.63 × 10^−4^	1.84 × 10^−2^
positive regulation of response to stimulus (GO:0048584)	0	7.48	−	<0.01	1.03 × 10^−3^	1.95 × 10^−2^
dicarboxylic acid transport (GO:0006835)	3	0.17	+	17.46	1.06 × 10^−3^	1.99 × 10^−2^
regulation of response to stimulus (GO:0048583)	3	13.76	−	0.22	1.07 × 10^−3^	1.99 × 10^−2^
fatty acid metabolic process (GO:0006631)	7	1.52	+	4.6	1.11 × 10^−3^	2.05 × 10^−2^
regulation of cellular metabolic process (GO:0031323)	14	30.05	−	0.47	1.17 × 10^−3^	2.14 × 10^−2^
glutathione metabolic process (GO:0006749)	4	0.44	+	9.05	1.37 × 10^−3^	2.45 × 10^−2^
fatty acid catabolic process (GO:0009062)	4	0.44	+	9.05	1.37 × 10^−3^	2.43 × 10^−2^
regulation of cellular biosynthetic process (GO:0031326)	9	22.27	−	0.4	1.66 × 10^−3^	2.93 × 10^−2^
regulation of biosynthetic process (GO:0009889)	9	22.34	−	0.4	1.66 × 10^−3^	2.91 × 10^−2^
cellular metabolic process (GO:0044237)	89	65.85	+	1.35	1.67 × 10^−3^	2.90 × 10^−2^
cellular component organization (GO:0016043)	50	32.21	+	1.55	1.77 × 10^−3^	3.04 × 10^−2^
mitochondrial transport (GO:0006839)	4	0.53	+	7.58	2.50 × 10^−3^	4.18 × 10^−2^
macromolecule metabolic process (GO:0043170)	41	61.45	−	0.67	2.64 × 10^−3^	4.37 × 10^−2^

**Table 2:** List of primary antibodies used with corresponding dilution and antibodies manufacturer’s catalogue number.

Primary Antibodies	Dilution	Source	Identifier
GSTM1	1:2000	Santa Cruz	Cat # sc-517262
GSTA1/2	1:2000	Santa Cruz	Cat # sc-398714
PSMA6	1:20,000	Abcam	Cat # 3109377
PSMD12	1:2000	Santa Cruz	Cat # sc-393401
PFK-1	1:2000	Santa Cruz	Cat # sc-166722
PKM2	1:1000	Santa Cruz	Cat # sc-135048
LMP2	1:2000	ThermoFisher Scientific	Prod # PA1-1960

## Data Availability

Data used and analyzed in the current study are available within the article and Supplementary Materials.

## Supplementary Materials

The following supporting information can be downloaded at https://www.mdpi.com/article/10.3390/ijms242015266/s1.
