# Supplementary material for: Mitochondrial and Proteasome Dysfunction Occurs in the Hearts of Mice Treated with Triazine Herbicide Prometryn
Source: Int J Mol Sci. 2023 Oct 17;24(20):15266. doi: 10.3390/ijms242015266 (PMC10607192; doi:10.3390/ijms242015266)
Supplement: Supplementary file 1 [file ijms-24-15266-s001.zip › Supplementary Figures Prometryn AG.pdf]

SUPPLEMENTARY FIGURES

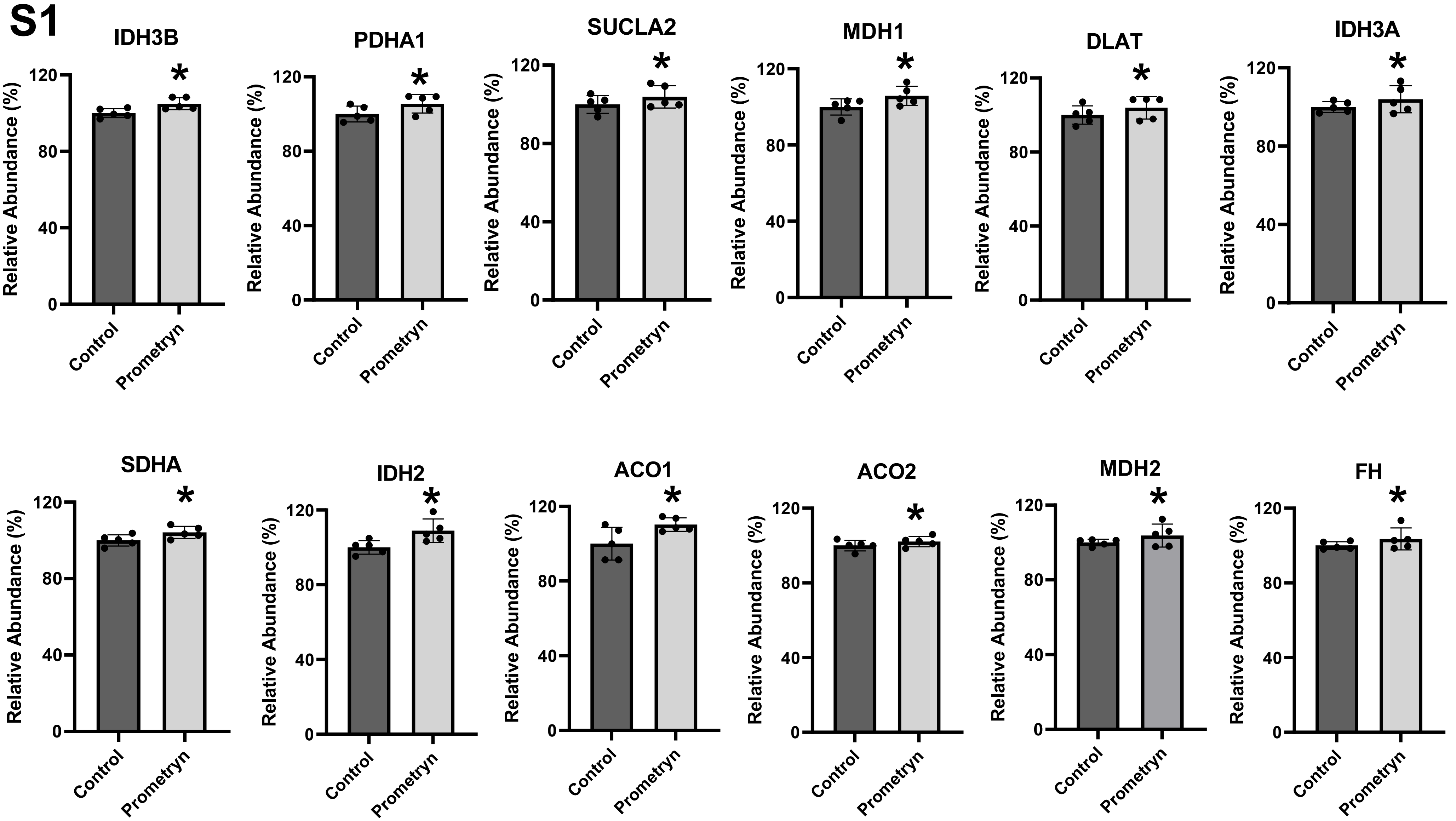
S1
IDH3B
PDHA1
SUCLA2
MDH1
DLAT
IDH3A
SDHA
IDH2
ACO1
ACO2
MDH2
FH
Relative Abundance (%)
0
40
80
120
Control
Prometryn
*

Supplemental Table S1. Table showing all proteins quantified in heart from vehicle and prometryn-treated mice.

Supplemental Figure S1. Abundance level of proteins involved in tricarboxylic acid cycle. Prometryn treatment increased the protein abundance of enzymes involved in tricarboxylic acid cycle (TCA): Isocitrate Dehydrogenase (NAD(+)) 3 Non-Catalytic Subunit Beta (IDH3B), pyruvate dehydrogenase E1 subunit alpha 1 (PDHA1), Succinate-CoA Ligase ADP-Forming Subunit Beta (SUCLA2), Malate Dehydrogenase 1 (MDH1), dihydrolipoamide S-acetyltransferase (DLAT), Isocitrate dehydrogenase [NAD] subunit alpha, mitochondrial (IDH3A), Succinate Dehydrogenase Complex Flavoprotein Subunit A (SDHA), Isocitrate dehydrogenase type 2 (IDH2), Aconitase 1 (ACO1), Aconitase 2 (ACO2), Malate Dehydrogenase 2 (MDH2), and fumarate hydratase (FH) as obtained by mass spectrometry. Value are mean ± SD; n = 5 per group. $^{*}p < 0.05$.

Supplemental Figure S2. Abundance level of proteins related to fatty acid metabolism in mice heart. Changes in protein abundance level of Electron Transfer Flavoprotein Dehydrogenase (ETFDH), Electron Transfer Flavoprotein Subunit Beta (ETFB), ATP Binding Cassette Subfamily D Member 3 (ABCD3), Hydroxyacyl-CoA Dehydrogenase Trifunctional Multienzyme Complex Subunit Alpha (HADHA), Carnitine Palmitoyltransferase 1B (CPT1B), Solute Carrier Family 25 Member 20 (SLC25A20), Enoyl-CoA Delta Isomerase 2 (ECI2), 2,4-Dienoyl-CoA Reductase 1 (DECR1), 3-Ketoacyl-CoA thiolase, mitochondrial (ACAA2), Enoyl-CoA Hydratase, Short Chain 1 (ECHS1), 17- β -Hydroxysteroid dehydrogenase type 10 (HSD17B10), and Fatty Acid Binding Protein 5 (FABP5) obtained by mass spectrometry. Value are mean ± SD; n = 5 per group. $^{*}p < 0.05$.

Supplemental Figure S3. Prometryn treatment induced heat-shock protein response to oxidative stress. Mass spectrometry revealed an increase in the abundance level of Heat Shock Protein Family A (Hsp70) Member 9 (HSPA9), Heat Shock Protein Family A (Hsp70) Member 4 (HSPA4), Heat Shock Protein Family B (Small) Member 1 (HSPB1), Heat Shock Protein Family D (Hsp60) Member 1 (HSPD1), and TNF receptor associated protein 1 (TRAP1), which belongs to the heat-shock protein that are cells in response to exposure to stressful conditions and they chaperone DNA repair. Value are mean ± SD; n = 5 per group. $^{*}p < 0.05$.

Supplemental Figure S4. Abundance level of apoptotic mitochondrial related protein in mice heart. Prometryn decreased the abundance level of Apoptosis Inducing Factor Mitochondria Associated 1 (AIFM1) and Mitochondrial Carrier 2 (MTCH2) obtained from mass spectrometry. Value are mean ± SD; n = 5 per group. $^{*}p < 0.05$.

Supplemental Figure S5. Heatmap representation showing sample abundances for 28 significantly altered proteins in control and prometryn-treated male mice heart. n = 5 per group.

S2

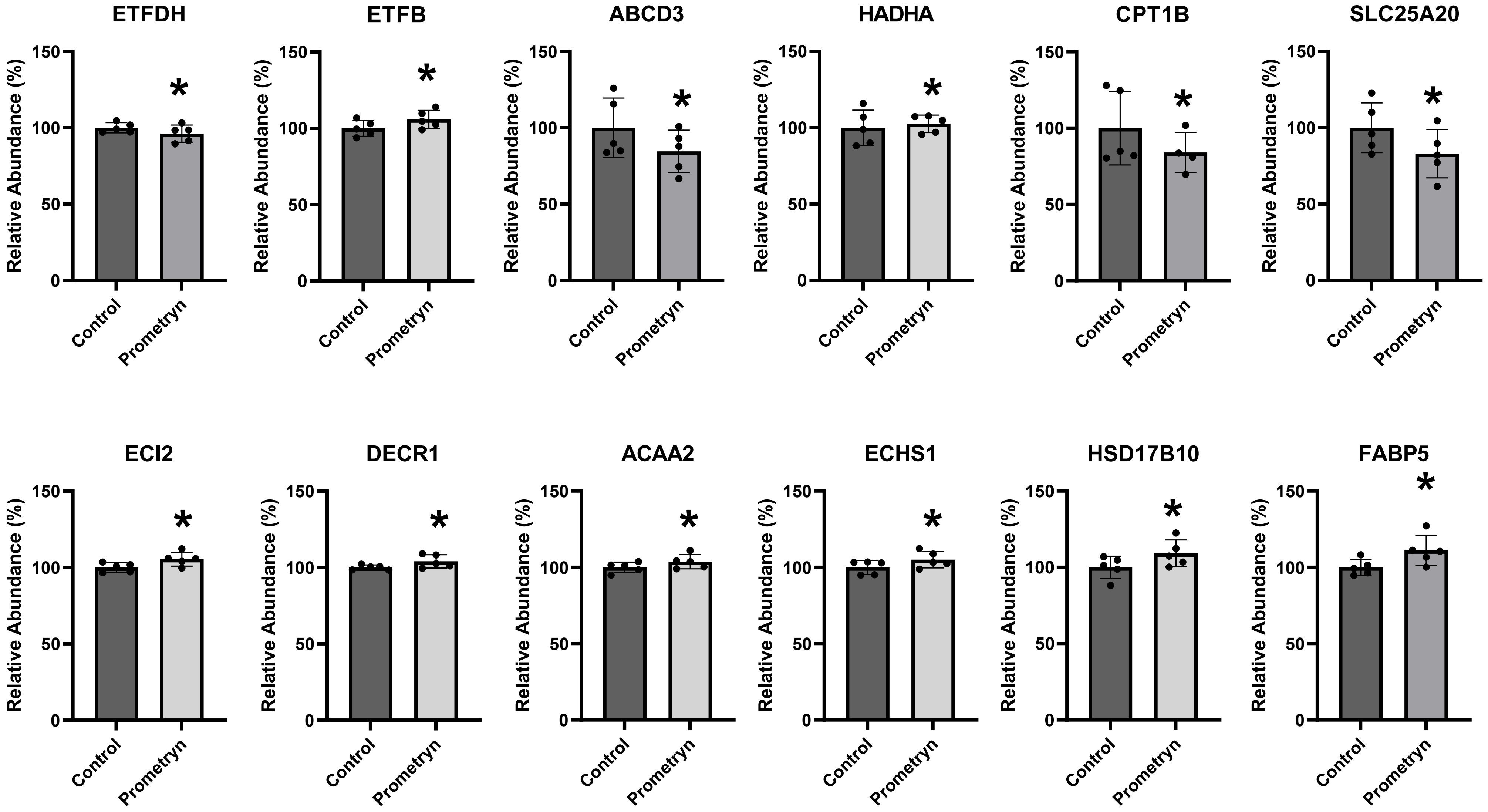
ETFDH
ETFB
ABCD3
HADHA
CPT1B
SLC25A20
ECI2
DECR1
ACAA2
ECHS1
HSD17B10
FABP5
Relative Abundance (%)
0
50
100
150
Control
Prometryn
*

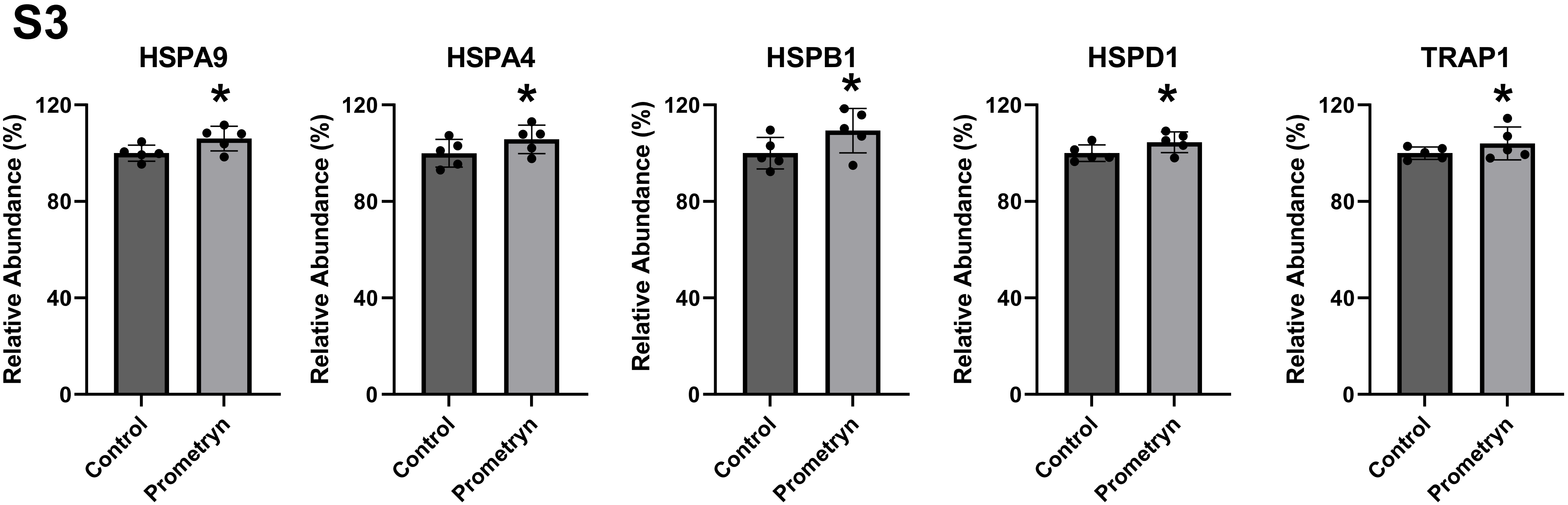
S3
HSPA9
HSPA4
HSPB1
HSPD1
TRAP1
Relative Abundance (%)
0
40
80
120
*
Control
Prometryn

S4

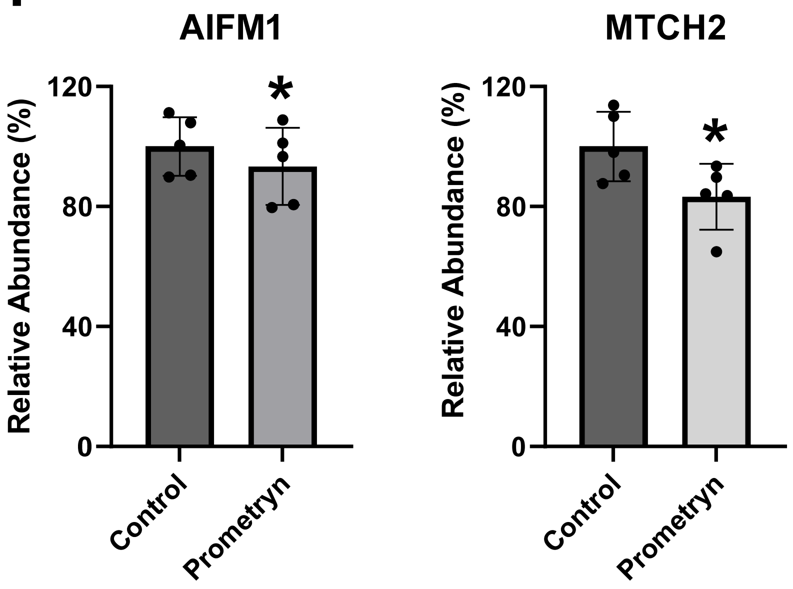

AIFM1
MTCH2
Relative Abundance (%)
120
80
40
0
*
Control
Prometryn

S5

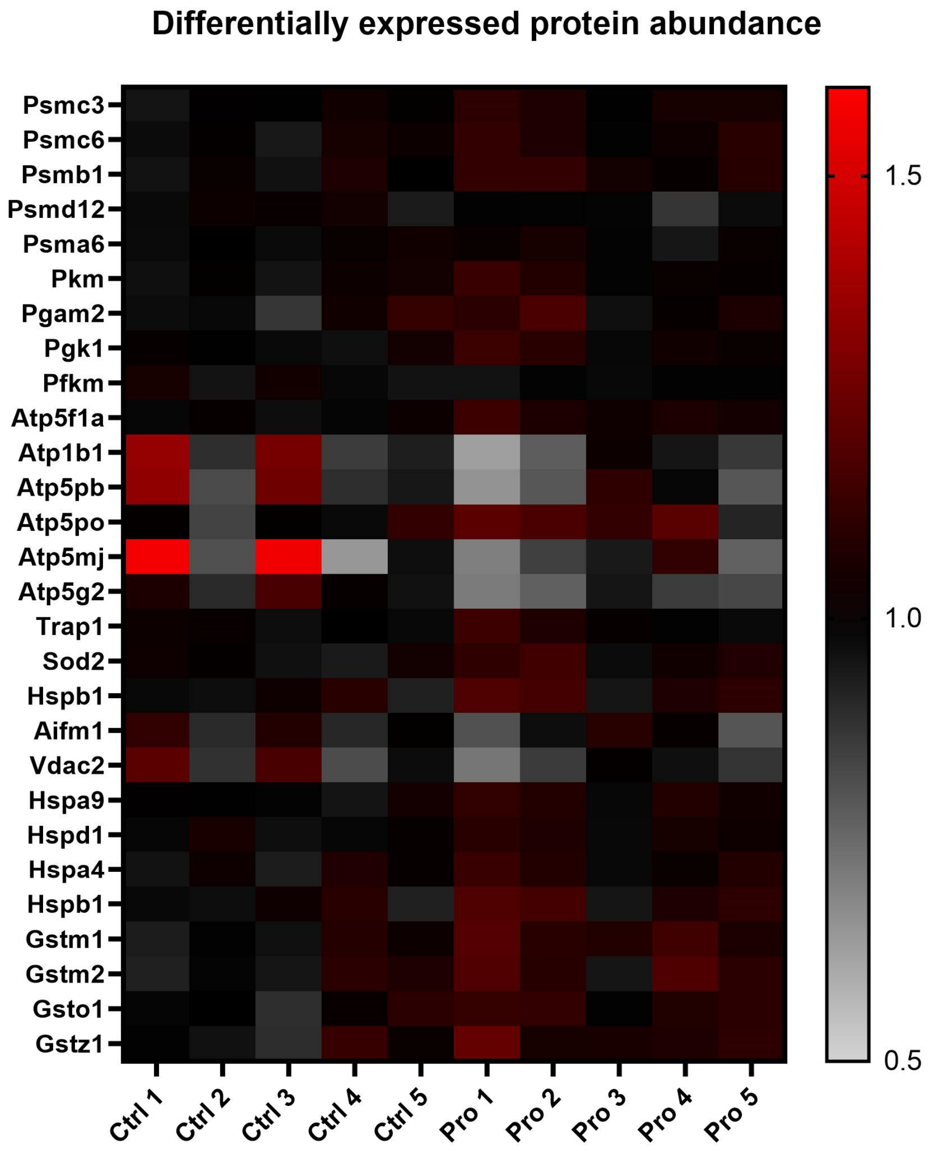
Differentially expressed protein abundance
Psmc3
Psmc6
Psmb1
Psmd12
Psma6
Pkm
Pgam2
Pgk1
Pfkm
Atp5f1a
Atp1b1
Atp5pb
Atp5po
Atp5mj
Atp5g2
Trap1
Sod2
Hspb1
Aifm1
Vdac2
Hspa9
Hspd1
Hspa4
Hspb1
Gstm1
Gstm2
Gsto1
Gstz1
Ctrl 1
Ctrl 2
Ctrl 3
Ctrl 4
Ctrl 5
Pro 1
Pro 2
Pro 3
Pro 4
Pro 5
1.5
1.0
0.5
